# Metabolites from *Streptomyces aureus* (VTCC43181) and Their Inhibition of *Mycobacterium tuberculosis* ClpC1 Protein

**DOI:** 10.3390/molecules29030720

**Published:** 2024-02-04

**Authors:** Thao Thi Phuong Tran, Ni Ngoc Thi Huynh, Ninh Thi Pham, Dung Thi Nguyen, Chien Van Tran, Uyen Quynh Nguyen, Anh Ngoc Ho, Joo-Won Suh, Jinhua Cheng, Thao Kim Nu Nguyen, Sung Van Tran, Duc Minh Nguyen

**Affiliations:** 1 Institute of Chemistry, Vietnam Academy of Science and Technology (VAST),18 Hoang Quoc Viet Road, Cau Giay, Hanoi 10000, Vietnam; ninhptvh@gmail.com (N.T.P.); nguyenthidungeon@gmail.com (D.T.N.); tvchien2015@gmail.com (C.V.T.); tranvansungvhh@gmail.com (S.V.T.); 2 Faculty of Chemistry, Graduate University of Science and Technology, VAST, 18 Hoang Quoc Viet Road, Cau Giay, Hanoi 10000, Vietnam; huynhthingocni0387@gmail.com; 3 Faculty of Natural Sciences, Phu Yen University, 01 Nguyen Van Huyen Road, Tuy Hoa City 56000, Vietnam; 4 Institute of Microbiology and Biotechnology, Vietnam National University Hanoi, 44, Xuan Thuy Road, Cau Giay, Hanoi 10000, Vietnam; uyennq@vnu.edu.vn; 5 Institute of Biotechnology, Vietnam Academy of Science and Technology (VAST), 18 Hoang Quoc Viet Road, Cau Giay, Hanoi 10000, Vietnam; hongocanh1612@gmail.com; 6 Center for Nutraceutical and Pharmaceutical Materials, Myongji University, Yongin 17058, Republic of Koreajhcheng1689@gmail.com (J.C.); 7 Faculty of Biology, University of Natural Sciences, Vietnam National University, Hanoi, 334 Nguyen Trai Road, Thanh Xuân, Hanoi 10000, Vietnam; 8 Institute of Genome Research, Vietnam Academy of Science and Technology (VAST), 18 Hoang Quoc Viet Road, Cau Giay, Hanoi 10000, Vietnam

**Keywords:** *Streptomyces aureus*, nocardamin, halolitoralin A, pleurone, ClpC1 protein, *Mycobacterium tuberculosis*

## Abstract

Tuberculosis is one of the most common infectious diseases in the world, caused by *Mycobacterium tuberculosis*. The outbreak of multiple drug-resistant tuberculosis has become a major challenge to prevent this disease worldwide. ClpC1 is a Clp ATPase protein of *Mycobacterium tuberculosis*, functioning as a chaperon when combined with the Clp complex. ClpC1 has emerged as a new target to discover anti-tuberculosis drugs. This study aimed to explore the ClpC1 inhibitors from actinomycetes, which have been known to provide abundant sources of antibiotics. Two cyclic peptides, including nocardamin (**1**), halolitoralin A (**3**), and a lactone pleurone (**2**), were isolated from the culture of *Streptomyces aureus* (VTCC43181). The structures of these compounds were determined based on the detailed analysis of their spectral data and comparison with references. This is the first time these compounds have been isolated from *S. aureus.* Compounds **1**–**3** were evaluated for their affection of ATPase activity of the recombinant ClpC1 protein. Of these compounds, halolitoralin A (**1**), a macrocyclic peptide, was effective for the ATPase hydrolysis of the ClpC1 protein.

## 1. Introduction

Today, tuberculosis (TB) is one of the fatal infectious diseases in humans caused by Mycobacteria tuberculosis that killed more than one billion people over the past 200 years [1,2]. In 2021, there were an estimated 1.6 million deaths and 10.6 million new TB infectious cases, of which about 450,000 new cases were rifampicin-resistant [3]. SARS and other viral infections can cause the recurrence of TB in affected patients [4]. The COVID-19 pandemic resulted in an increase in the number of undiagnosed TB cases. As a consequence, the number of people dying from TB has increased [5]. It was estimated that about 400,000 people died from tuberculosis because of the added strain from COVID-19 on healthcare systems worldwide [6]. In Vietnam, there were about 174,000 new cases and 11,000 deaths in 2021 [7]. Antibiotics to treat TB have been available for over 50 years. However, multidrug-resistant TB (MDR) and extensive drug-resistant TB (XDR) are still serious public health issues [8]. According to the Vietnamese National Tuberculosis Control Program report, 8400 new cases of rifampicin-resistant and multidrug-resistant TB were found in 2021 [9]. Thus, the development of new antibiotics that possess new mechanisms of action is needed to treat TB.

The Clp proteins play a crucial role in the pathogenicity and survival of many general pathogenic microorganisms, including *Mycobacterium tuberculosis*. Clp proteins of *M. tuberculosis* are composed of Clp protease (ClpP1 and ClpP2) and Clp ATPase (ClpC1 and ClpX). Unlike many other Clp mycobacteria proteins, ClpC1 in *M. tuberculosis* has a highly conserved sequence, acting as a chaperon in the cell when combined with the Clp complex [10]. If the activation of ClpC1 is disturbed or broken down, the protein degradation in the cells will be reduced or stopped altogether [11,12]. Due to its important role, the ClpC1 protein, which could be produced by recombination on *Escherichia coli* in a laboratory, has been considered a new target in the development of anti-tuberculosis agents [10,13]. Recently, naturally cyclic peptides isolated from actinomycetes and fungi, such as cyclomarin, ecumicin, lassomycin, and rufomycin, have been shown to affect the ATPase activity of ClpC1 [10,14,15]. Cyclic peptides with properties such as low toxicity, good binding affinity, and target selectivity are potential sources for screening a wide range of biological activities [16]. Cyclomarin A is a cyclopeptide found in the marine actinomycetes *Streptomyces* spp. CNB-982. Recently, this compound was discovered to have anti-tuberculosis activity through the mechanism of targeting the regulatory protein ClpC1 of tuberculosis bacteria [17]. Cyclomarin A includes seven amino acids, with two basic amino acids (alanine and valine) and the remaining five uncommon amino acids. Lassomycin was isolated from the actinomycete *Lentzea kentuckyensis* spp. IO0009804. This compound consists of 16 amino acids and is composed of a cyclic chain of 8 N-terminal amino acids attached to the straight chain by a bond between the N-terminal amino group and the carboxyl group of Asp8 [18]. Ecumicin is a macrocyclic peptide isolated from actinomycetes *Nonomurae* spp. MJM5123. Eucumicin includes 13 amino acids, containing standard and methoxylated amino acids in the cyclic peptide chain. Hanki Lee and his colleagues studied the mechanism of the ecumicin effect on ClpC1 and reported that this compound binded to the allosteric site of ClpC1, causing a change in configuration, thereby affecting proteolysis of ClpC1/CplP1/ClpP2 [19].

*Streptomyces aureus* is a species of actinomycete belonging to the genus *Streptomyces*. Its spores are spherical or oval in shape, and the average diameter is about 0.9 μ in width and 1.2 μ in length [20]. *S. aureus* lives in soil environments, is cultured at appropriate conditions of 28 °C, and does not grow at 50 °C. The morphological characteristics of the *S. aureus* depend on different nutritional environments. A previous study reported that many active secondary metabolites were produced by this actinomycete strain. Alkaloid azirinomycin and 3-methyl-2H-azirine-2-carboxylic acid isolated from *S. aureus* exhibited broad-spectrum antibiotic activity in vitro against Gr (+) and Gr (-) microbacteria [20,21]. Antibiotics type macrotetrolide, including tetranactin, dinactin, and trinactin, were also isolated from *S. aureus* [22]. In addition, the antibiotic group manumycin, including colabomycin E-G and dinorlabomycin A, dinorlabomycin E was isolated from the mutant strain *S. aureus* SOK1/5-04 colC3C4C5 [23]. The adenosine derivatives aureonuclemycin A and aureonuclemycin B were isolated from *S. aureus* SPRI-371 from the soil environment in Jiangsu province, China [24].

Within the framework of searching for the metabolites from actinomycetes targeting the ATPase hydrolysis activity of ClpC1, two cyclic peptides including nocardamin **(1)**, halolitoralin A (**3**), and a lactone pleurone (**2**) from the culture solution of *Streptomyces aureus* VTCC43181 were isolated and structurally elucidated. These compounds were evaluated for the ATPase hydrolysis activity of ClpC1, a recombinant protein from *Escherichia coli* produced in our laboratory. Compound **3** showed the effect on the ATPase hydrolysis activity of ClpC1, acting similar way to the antibiotic eucumicin.

## 2. Results and Discussion

### 2.1. Identification of the Strain VTCC43181

**Figure 1:**
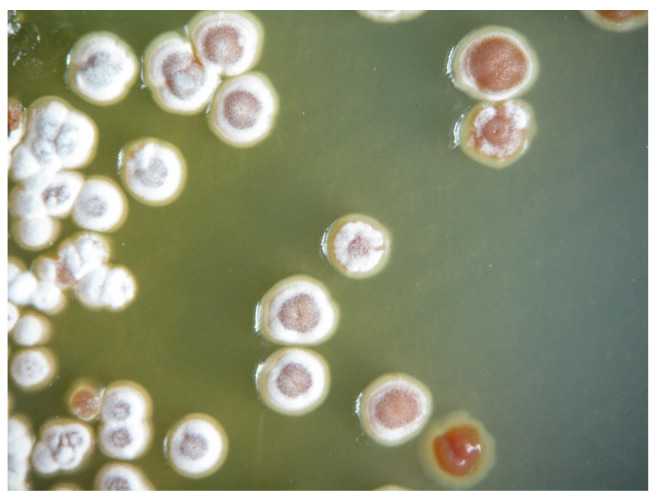
Colony morphology of *Streptomyces aureus* VTCC43181.

The strain VTCC43181 was identified as belonging to the genus *Streptomyces* based on its preliminary observation of morphology. The colonies are convex and white, with straight chains of spores. The 16S rRNA sequence of strain VTCC43181 was 100% homologous to *Streptomyces aureus* NBRC 100912 (Figure 1). The 16S rDNA sequence and the phylogenetic tree of the strain are shown in the Supporting information (Figures S1 and S2).

### 2.2. Chemistry

Compounds **1**–**3** were obtained from the ethyl acetate extract (4.5 g) of the culture solution from the strain *Streptomyces aureus* VTCC43181 using different chromatographic methods.

**Figure 2:**
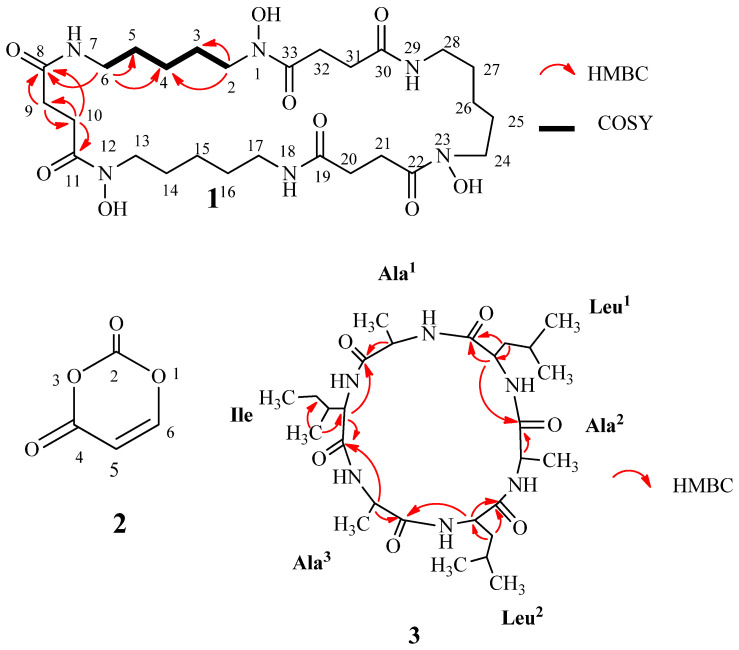
The structures, HMBC, and COSY correlations of the isolated compounds (**1–3**) from *Streptomyces aureus*.

Compound **1** (7 mg) was afforded after purification using Sephadex and silica gel column chromatography, followed by thin-layer chromatography. This compound was obtained as a colorless powder. The HRESI-MS spectrum of **1** showed molecular ion peaks at *m/z* 601.3527 (calcd for C_27_H_49_N_6_O_9_ 601.3561 [M+H]^+^) and 623.3353 (calcd for C_27_H_48_N_6_O_9_Na 623.3380 [M+Na]^+^). The ^1^H NMR spectrum of compound **1** displayed seven methylene protons at δ_H_ 3.62 (4H, t, *J* = 6.5, H-2), 3.19 (4H, t, *J* = 6.5, H-6), 2.79 (4H, t, *J* = 6.5, H-9), 2.49 (4H, t, *J* = 6.5, H-10), 1.65 (4H, m, H-3), 1.53 (4H, m, H-5), and 1.35 (4H, m, H-4). The ^13^C-NMR spectrum indicated seven methylene carbons at δ_C_ 49.1 (C-2), 40.1 (C-6), 28.9 (C-9), 31.6 (C-10), 29.7 (C-5), 27.1 (C-3), 24.6 (C-4), and two carbonyl carbons at δ_C_ 174.7 (C-8), 174.5 (C-11). In the 1H 1H COSY spectrum, crossing correlations of H-6 (δ_H_ 3.19 with H-5 (δ_H_ 1.53), H-5 (δ_H_ 1.53 with H-4 (δ_H_ 1.35), H-4 (δ_H_ 1.35) with H-3 (δ_H_ 1.65), and H-3 (δ_H_ 1.65) with H-2 (δ_H_ 3.62) were observed.The HMBC spectrum confirmed correlations from H-2 (δ_H_ 3.62) to C-3 (δ_C_ 27.1) and C-4 (δ_C_ 24.6), H-6 (δ_H_ 3.19) to C-4 (δ_C_ 24.6), C-5 (δ_C_ 29.7) and C-8 (δ_C_ 174.7), H-9 (δ_H_ 2.79) to C-8 (δ_C_ 174.7), C-10 (δ_C_ 31.6) and C-11 (δ_C_ 174.5), H-10 (δ_H_ 2.49) to C-8 (δ_C_ 174.7), and C-9 (δ_C_ 28.9) (Figure 2). A combination of NMR and HRESI-MS data suggested compound **1** as a macrocyclic hydroxamate. By comparison with the reported data, compound **1** was assigned as nocardamin, a cyclic trimer of N-hydroxy-N′-succinylcadaverine [25,26]. Nocardamin was a hydroxamate siderophore and was first isolated from the *Nocardia* strain [27]. This compound showed siderophore activity and antimalarial activity [25].

Compound **2** (3 mg) was yielded after column chromatography on normal-phase silica gel and the Sephadex LH20 column. Compound **2** (3 mg) was obtained as a white powder. The (+)-ESI-MS spectrum of **2** showed a molecular ion peak at *m/z* 119 [M+Na-H_2_O]^+^. The ^1^H NMR spectrum exhibited two signals of olefinic protons at δ_H_ 5.43 (1H, d, *J* = 7.5 Hz, H-5) and 7.39 (1H, d, *J* = 8.0 Hz, H-6). The ^13^C NMR spectrum showed signals of two carbonyls at δ_C_ 164.4 (C-4), 151.7 (C-2) and two olefinic carbons at δ_C_ 100.0 (C-5), and 142.6 (C-6). The NMR data of compound **2** indicated a lactone ring structure consisting of four carbon atoms and two oxygen atoms. Compared with the published data [28], **2** was determined as pleurone (4H-1,3-dioxine-2,4-dione) (Figure 2). Pleurone possesses antioxidant activity and leukocyte inhibitory effects [28]. In addition, it also exhibited inhibitory activity against HCV NS3 protease with an IC_50_ value of 78.9 μM [29] and was considered a potent compound for the development of anti-HCV (hepatitis C virus) drugs [29].

Compound **3** (12 mg) was obtained as a white solid. This compound was afforded after two reverse-phase column chromatography. The HRESI-MS spectrum of **3** showed molecular ion peaks at *m/z* 553.3730 (calcd for C_27_H_49_N_6_O_6_ 553.3714 [M+H]^+^) and 575.3548 (calcd for C_27_H_48_N_6_O_6_Na 575.3533 [M+Na]^+^). In the ^1^H NMR spectrum, ten signals of protons appeared at δ_H_ 3.57–3.54 (2H, m, α-H of Leu^1^ & Leu^2^), 3.50 (1H, d, *J* = 3.5, α-H of Ile), 3.43 (3H, d, *J* = 4.5, α-H of Ala^1^, Ala^2^, and Ala^3^), 1.97–1.95 (1H, m, β-H of Ile), 1.83–1.77 (2H, m, γ-H of Leu^1^, and Leu^2^), 1.83–1.59 (4H, m, β-H of Leu^1^, and Leu^2^), 1.66–1.59 (2H, m, γ-H of Ile), 1.08–1.07 (3H, d, J = 7.0, β-H of Ala^3^), 1.05–1.01 (9H, m, β-H of Ala^1^, and Ala^2^, β′-H of Ile), and 1.00–0.96 (15H, m, δ-H of Leu^1^, and Leu^2^, δ-H of Ile). The ^13^C NMR spectrum showed signals of nine methyls, three methylenes, nine methenyls, and six carbonyls at δ_C_ 176.0 (C=O, Leu^2^), 175.2 (C=O, Leu^1^), 174.5 (C=O, Ala), and 174.0 (C=O, Ile). The NMR spectral data of compound **3** showed characteristics of cyclic hexapeptide. The HMBC correlations between H-*α* (Ala) (δ_H_ 3.43) with C=O (Ala) (δ_C_ 174.5) and C=O (Leu) (δ_C_ 175.2); H-*β* (Ala) (δ_H_ 1.08–1.01) and C-*α* (Ala) (δ_C_ 61,8); H-*α* (Leu) (δ_H_ 3.57–3.54) and C=O (Ala) (δ_C_ 174.5) and C=O (Leu) (δ_C_ 175.2); H-*β* (Leu) (δ_H_ 3.57–3.54) with C=O (Leu) (δ_C_ 175.2) and C-*α* (Leu) (δ_C_ 54.7); H-*α* (Ile) (δ_H_ 3.50) with C=O (Ala) (δ_C_ 174.5) and C=O (Ile) (δ_C_ 174,0), H-*β*′ (Ile) (δ_H_ 1.05–1.01) and C-*α* (Ile) (δ_C_ 60.9), and C-*γ* (Ile) (δ_C_ 25.9) were observed (Fiugre 2). The peptide chain was further confirmed by the appearance of the fragment signals in -IDA TOF MS/MS at *m/z* 340 [Ala-Leu-Ala-Leu-CO]^−^, 255 [Ala-Leu-Ala]^−^, and 227 [Ala-Leu-Ala-CO]^−^. By analysis of the spectral data and comparing it with the published data, compound **3** was determined as halolitoralin A, a hexapeptide containing three Ala, two Leu, and one Ile unit [30,31]. Halolitoralin A was isolated from *Halobacillus litoralis* YS3106 of marine origin (Huanghai Sea, China) [30]. This compound showed potent antimicrobial activity, anthelmintic activity [31], and antitumor activities in vitro [30].

### 2.3. Affection of the Isolated Compounds to ATPase Activity of ClpC1

**Figure 3:**
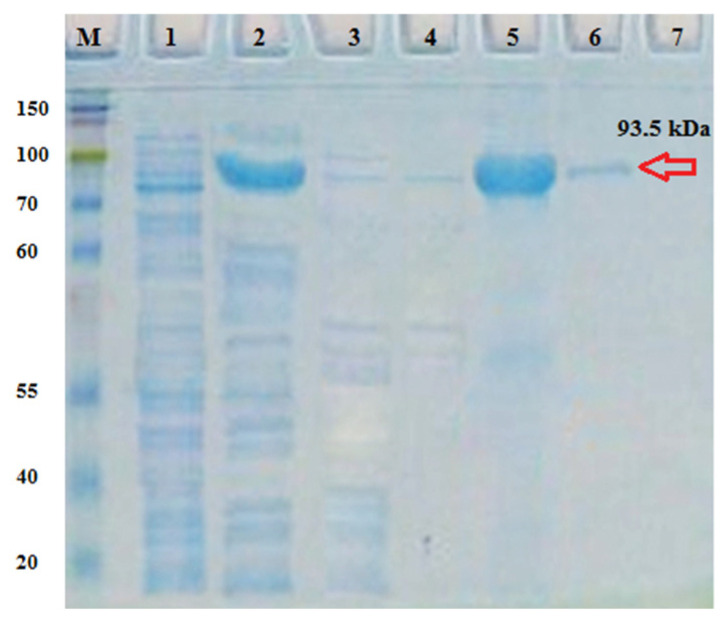
Purification of recombinant *Mycobacterium tuberculosis* ClpC1: electrophoretic analysis of affinity chromatography using Ni-TED resin. M: maker; 1: non-induced pET28a(+)/ClpC1; 2: induced pET28a(+)/ClpC1; 3, 4: washing fractions; and 5, 6, 7: elution fractions.

**Figure 4:**
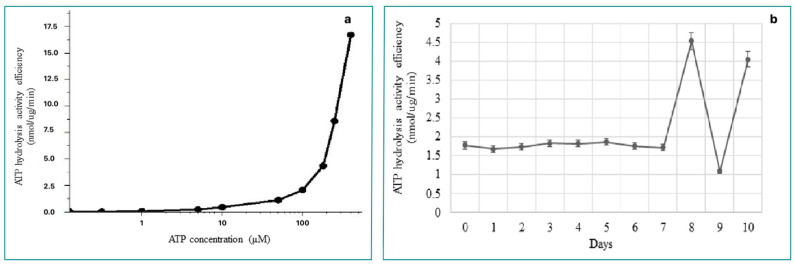
(**a**) Evaluation of ATPase activity. (**b**) Evaluation of ATPase stability.

The purification of the ClpC1 protein was conducted using the Ni-TED column (Figure 3). The recombinant ClpC1 protein was obtained with an approximate molecular weight of 93.5 kDa. The ATP hydrolysis activity and the stable level of recombinant ClpC1 protein were monitored throughout the experiment. To determine if our recombinant ClpC1 possessed inherent ATPase activity, its enzymatic hydrolysis of radioactive ATP was analyzed, and the generated radioactive inorganic phosphate was quantified. ATP hydrolysis activity via ClpC1 was recorded at 10 μM, following the gradual increase of the ATP concentration in the reaction (Figure 4a). Therefore, our recombinant ClpC1 protein exhibited full efficiency in ATP hydrolysis. The stability of ATPase activity in ClpC1 protein was also examined. As a result, the ATPase activity remained at its original properties for seven days (Figure 4b) but was largely changed and unstable by day 8.

**Figure 5:**
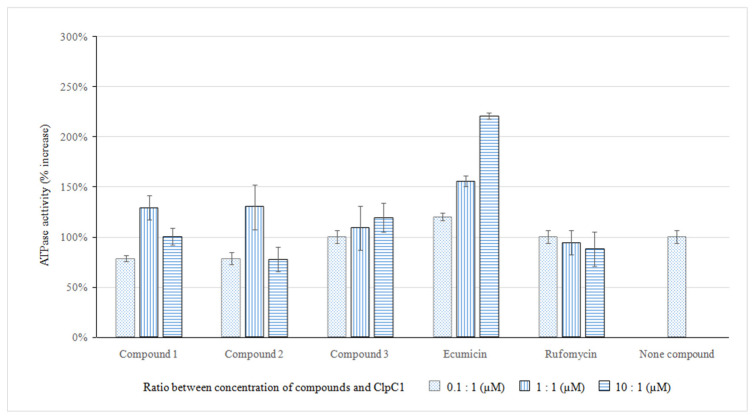
Affection of compounds **1**, **2**, **3**, rufomycin, and ecumicin to ATPase activity.

Several compounds, such as ecumicin and rufomycin, have been reported to inhibit ClpC1 through the effection of ATP hydrolysis function [10,14,15]. Therefore, these compounds were used as positive controls in this study. Different concentrations of compounds **1**, **2,** and **3** (0.1 µM, 1.0 µM, and 10.0 µM) were evaluated and optimized for the ATPase activity of the ClpC1 protein. The results showed that ATPase activity increased gradually with the concentration of compound **3** (Figure 5). It was observed that compound **3** was effective for the ATP hydrolysis of ClpC1 protein, similarly to ecumicin, but with lower ATP hydrolysis affection, while compounds **1** and **2** did not affect the ATPase activity of ClpC1. The above results suggested that compound **3** directly targeted the ClpC1 protein of *M. tuberculosis* through the affection of ATPase activity. Comparing the structure of compound **3** to those of potential macrocyclic compounds that inhibited ClpC1, such as ecumicin, rufomycin, lassomycin, compound **3** was a hexapeptide with a simpler structure, suggesting the total synthesis and structural modification to enhance the ClpC1 inhibition.

## 3. Materials and Methods

### 3.1. Sample Collection

*Streptomyces aureus* VTCC43181 was cultured and provided by the National Microbial Gene Resource Center, Institute of Microbiology and Biotechnology, Hanoi National University. The soil sample was collected in Langbiang Mountain, Lac Duong district, Lam Dong province, Vietnam.

### 3.2. Equipment and Chemicals

NMR nuclear magnetic resonance spectra were recorded with a Bruker Avance 500 NMR spectrometer (Bremen, Germany). ESI-MS mass spectra were measured on an Agilent LC-MSD-Trap SL. The HR-ESI-MS high-resolution mass spectrum was measured on a Varian (California, USA) FT-ICR-MS instrument. Thin-layer chromatography (TLC) was performed on Merk 60 F254 silica gel plates. Column chromatography was conducted with silica gel (40–63 μm particle size), diaion HP-20, RP-18, and Sephadex LH-20 (Aldrich, St. Louis, MO, USA). Other chemicals were purchased from Merck and used without purification unless otherwise needed. TLC was sprayed with a Dragendorff reagent and vanillin/H_2_SO_4_ reagents, visible by heating at 80–100 °C. Equipment for actinomycete cultivation: sterile cabinet for culture plates (Nuaire, Caerphilly, PA, USA), incubator (Binder, Tuttlingen, Germany), autoclave (Hirayama, Kasukabe-Shi Saitama, Japan), incubation shaker (Certomat BS-1, Sartonius, Goettingen, Germany), refrigerator (−80 °C, Nuaire, Caerphilly, PA, USA), refrigerated centrifuge (Centrifuge C30P Sartorius Group, Goettingen, Germany), and pipette Pasteur (Thomas Scientific, Swedesboro, NJ, USA); automatic pipette; technical scale (Precisa XB 1200C, Dietikon, Switzerland), analytical balance (Shimadzu AY 120, Kyoto, Japan), pH meters (Hanna instrument, Tokyo, Japan), optical microscope (Olympus CX21, Tokyo, Japan), and fermentor (B. E. Marubishi, Tokyo, Japan).

### 3.3. Isolation of the Actinomycete

The sample was dried and powdered, diluted to 10^−3^, 10^−4^ concentration, and spread onto a Petri dish. HV media (Humic acid 1 g, CaCO_3_ 0.02 g, FeSO_4_.7H_2_O 0.01g, KCl 1.7 g, MgSO_4_.7H_2_O 0.05 g, Na_2_HPO_4_ 0.5 g, B vitamins 5mL, Agar 16 g, distilled water 1L, itpH 7) and nalidixic acid (20 mg/L) supplemented with cycloheximide (50 mg/L) were used as media. The isolation plates were incubated at 28 °C for 15 days. The agar pieces were kept in tubes containing 20 % glycerol and kept at −80 °C for further study.

### 3.4. Identification of the Actinomycete

The identification and classification of actinomycete were determined based on the spore morphology of the colony, in combination with 16S rRNA gene sequencing. The primers pair 27F (5′-AGAGTTTGATCCTGGCTCAG-3) and 1492R (5′-GGTTACCTTGTTACGACTT-3) was used to amplify the 16S rRNA gene in the PCR method [32]. The EZ Taxon tool was used to detect the sequence of the 16S rRNA fragment. Reference sequences used in phylogenetic tree research were taken from data from DDBJ, EMBL, and GenBank. The phylogenetic tree was built with 1000 repetitions on MEGA software version 5.05.

### 3.5. Extraction and Isolation of the Compounds from Streptomyces aureus

The strain stored at −80 °C was transferred to a −20 °C refrigerator for 1 h, then to 4–8 °C for another one hour, and kept at room temperature for 5–10 min before being transferred to a culture medium. The isolated actinomycete was grown on a YS medium at 30 °C for 6 days. The uniformity of colony morphology and spore chain morphology were observed to ensure the purity of the strain before propagation. For the first propagation, the actinomycete biomass on the agar plate was collected and transferred to a 250 mL Erlenmeyer flask containing 125 mL of a YS medium, shaken at 160 rpm at 28–30 °C for 24 h. The culture from the first propagation was transferred to a 5 L Satorius culture apparatus containing 2.5 L of a sterile YS medium, followed by shaking for 24 h at 30 °C at 300 rpm, aeration 1 L/min. The purity and the density of actinomycete cells were checked after each propagation step. The seed liquid (2.5 L) was inoculated into a CPR fermenter containing 40 L of a sterilized YS medium. The fermentation broth (10 mL) was collected every 24 h to determine the purity and growth of actinomycete. The highest density of the strain culture was reached after 144 h (6 days). The cell residues were removed from the culture using centrifugation (10,000 rpm, 30 min). The filtrate (30 L) was extracted with EtOAc (3 × 24 h × 15 L). The EtOAc extracts were combined and the solvent was evaporated in vacuo to obtain a crude residue (4.5 g, CN), which was subjected to a Sephadex LH-20 column (MeOH/H_2_O 9:1) to yield six fractions (CN.1 to CN.6). Fraction CN.1 (2.1 g) was chromatographed on a silica gel column (EtOAc/MeOH 95:5→1:1) to obtain three fractions (CN.1.1 to CN.1.3). Fraction CN.1.1 (54 mg) was subjected to a silica gel column (EtOAc/MeOH 95:5→1:1) to obtain three fractions (CN.1.1.1 to CN.1.1.3). Fraction CN.1.1.3 (24 mg) was further purified using preparative thin-layer chromatography to yield compound **1** (7 mg). Fraction CN.4 (1.2 g) was subjected to a normal phase silica gel column (EtOAc/MeOH/H_2_O 9:1:0.1) to afford 10 fractions (CN4.1→CN4.10). Fraction CN4.1 (20 mg) was purified via a Sephadex LH-20 column (MeOH 100%) to afford compound **2** (3 mg). Fraction CN4.9 (50 mg) was separated on a reverse phase (RP) silica gel column (MeOH/H_2_O 6:4) to obtain four fractions (CN4.9.1→CN4.9.4). Fraction CN4.9.3 (37 mg) was purified via the RP column (MeOH/H_2_O 6:4) to give compound **3** (12 mg).

Nocardamin **(1):** Colorless, powder; C_27_H_48_N_6_O_9_; HRESI-MS (*m/z*): 601.3527 (calcd for C_27_H_49_N_6_O_9_ 601.3561 [M+H]^+^), 623.3353 (calcd for C_27_H_48_N_6_O_9_Na 623.3380 [M+Na]^+^); ^1^H NMR (δ_H_, *J*, 500 MHz, CD_3_OD): 3.62 (4H, t, *J* = 6.5, H-2), 3.19 (4H, t, *J* = 6.5, H-6), 2.79 (4H, t, *J* = 6.5, H-9), 2.49 (4H, t, *J* = 6.5, H-10), 1.65 (4H, m, H-3), 1.53 (4H, m, H-5), 1.35 (4H, m, H-4); ^13^C NMR (δ_C_, 125 MHz, CD_3_OD): 174.7 (C-8), 174.5 (C-11), 49.1 (C-2), 40.1 (C-6), 28.9 (C-9), 31.6 (C-10), 29.7 (C-5), 27.1 (C-3), and 24.6 (C-4).

Pleurone **(2):** White, powder; C_18_H_32_N_4_O_6_; (+) ESI-MS (*m/z*): 119 [M+Na-H_2_O]^+^; ^1^H NMR (δ_H_, *J*, 500 MHz, DMSO): 7.39 (1H, d, *J* = 8.0, H-6), 5.43 (1H, d, *J* = 7.5, H-5); ^13^C NMR (δ_C,_ 125 MHz, DMSO): 164.4 (C-4), 151.7 (C-2), 142.6 (C-6), and 100.0 (C-5).

Halolitoralin A **(3):** White, solid; C_27_H_48_N_6_O_6_; HR-ESI-MS (*m/z*): 553.3730 (calcd for C_27_H_49_N_6_O_6_ 553.3714 [M+H]^+^), 575.3548 (calcd for C_27_H_48_N_6_O_6_Na 575.3533 [M+Na]^+^); ^1^H NMR (δ_H_, *J*, 500 MHz, CD_3_OD): 3.57–3.54 (2H, m, *α*-H of Leu^1^, and Leu^2^), 3.50 (1H, d, *J* = 3.5, *α*-H of Ile), 3.43 (3H, d, *J* = 4.5, *α*-H of Ala^1^, Ala^2^, and Ala^3^), 1.97–1.95 (1H, m, *β*-H of Ile), 1.83–1.77 (2H, m, *γ*-H of Leu^1^, and Leu^2^), 1.83–1.59 (4H, m, *β*-H of Leu^1^, and Leu^2^), 1.66–1.59 (2H, m, *γ*-H of Ile), 1.08–1.07 (3H, d, J = 7.0, *β*-H of Ala^3^), 1.05–1.01 (9H, m, *β*-H of Ala^1^, and Ala^2^, *β*′-H of Ile), 1.00–0.96 (15H, m, δ-H of Leu^1^, and Leu^2^, δ-H of Ile); ^13^C NMR (δ_C_, 125 MHz, CD_3_OD): 176.0 (C=O, Leu^2^), 175.2 (C=O, Leu^1^), 174.5 (C=O, Ala), 174.0 (C=O, Ile), 61.8 (*α*-C, Ala^1^, Ala^2^, and Ala^3^), 60.9 (*α*-C, Ile), 54.7 (*α*-C, Leu^1^, and Leu^2^), 41.8 (*β*-C, Leu^1^, and Leu^2^), 37.7 (*β*-C, Ile), 25.9 (*γ*-C, Ile), 25.8 (*γ*-C, Leu^1^, and Leu^2^), 23.2 (*δ*-C, Leu^2^), 22.0 (δ-C, Leu^1^), 19.1 (*β*-C, Ala^3^), 17.7 (*β*-C, Ala^1^, and Ala^2^), 15.6 (*β*′-C, Ile), and 12.2 (*δ*-C, Ile).

### 3.6. Screening for ClpC1 Inhibitors

#### 3.6.1. Cloning, Expression and Purification Recombinant Protein ClpC1

The cloning, expression, and purification experiments utilized TOP10 and Rosetta 2 (DE3) strains of *Escherichia coli* (Novagen). The LB medium served as the culture medium supplemented with 50 µg/mL Kanamycin antibiotic. The vector pET28a(+) was employed for transforming the specific ClpC1 gene and expressing the recombinant ClpC1 protein. Protein expression was induced using IPTG (isopropyl 1-thio-*β*-D galactopyranoside-Sigma Corp, Tokyo, Japan) after reaching an optical density (O.D) of the culture at 600 nm, as described in the literature [33,34]. The recombinant ClpC1 protein was purified using a Ni-TED spin column (Macherey-Nagel). The insoluble fraction, soluble fraction, and eluted protein were analyzed using the SDS-PAGE Mini-PROTEAN Electrophoresis System (Bio-rad, Hercules, California, CA, USA) with a 12% gel. The gel was then incubated with a staining solution (EZ-Gel Staining solution, DOGEN) (Nagel, 2011). The protein concentration was determined prior to use.

#### 3.6.2. Measurement of Protein Concentration and Buffer Exchange

Protein concentration was determined using the Bradford method in a Microliter plate. Three dilutions of a protein standard (Bovine Serum Albumin-BSA), representative of the protein solution to be tested, were prepared. The linear range of the assay was 8 μg/mL to approximately 80 μg/mL. Protein solutions were typically assayed in triplicate, and absorbance was measured at 595 nm using an Infinite^®^ 200 PRO (Tecan, Männedorf, Switzerland) (Bradford, 1976). Elution was performed using a buffer containing 50 mM Tris-HCl, 100 mM KCl, 8 mM MgCl2, pH 7.5, with PD-10 Desalting Columns (GE Healthcare Bio-Sciences AB, Uppsala, Sweden).

#### 3.6.3. ATPase Activity Assay

ATPase activity of ClpC1 was determined by monitoring the released phosphate level using BIOMOL^®^ Green (Enzo Life Science, Farmingdale, NY, USA). The development of green color intensity was monitored via absorbance at 620 nm. The amount of phosphate released was converted using a phosphate standard curve. A graph of phosphate released versus ATP concentration was plotted with the OriginPro 8 program. The curve was fitted using Hill equations as follows: y = START + (END-START) × xn/(kn + xn) (Ito, Akiyama, 2005). Statistical analysis was performed with OriginPro8 and Excel software. Data were expressed as mean ± standard error (SD) or frequency (%). Ecumicin and rufomycin (provided by the Nutrition and Pharmaceutical Center, Yongin, Republic of Korea) were used as a positive control in this screening study [14,35,36].

## 4. Conclusions

In conclusion, to the best of our knowledge, nocardamin (**1**), pleurone (**2**), and halolitoralin A (**3**) were isolated and identified from the strain *Streptomyces aureus* VTCC43181 for the first time. The structures of these compounds were fully characterized using modern spectroscopic methods such as HR-ESIMS, 1D, and 2D NMR. Evaluation of ATPase activity of these compounds on the target recombinant protein ClpC1 led to the identification of the promising compound (halolitoralin A, compound **3**). This compound affected the ATPase activity of ClpC1 in a similar way to the standard compound ecumicin. Further research using molecular modeling is needed to study the mechanism and active sites of compound **3** on *M. tuberculosis* ClpC1 protein. A synthetic approach and structural modification of halolitoralin A are also necessary to conduct the ClpC1 inhibition SAR study. To scale up the amount of compound **3** for further study purposes, the fermentation process of *S. aureus* VTCC43181 on a large scale is currently being carried out in our laboratory.

## Data Availability

The data presented in this study are available upon request from the corresponding authors.

## Supplementary Materials

The following supporting information can be downloaded at https://www.mdpi.com/article/10.3390/molecules29030720/s1, Figures S1 and S2: 16S rDNA sequence and the phylogenetic tree of the strain. Figure S3: The structures, HMBC and COSY correlations of the isolated compounds (**1-3**) from *Streptomyces aureus* VTCC43181. Figures S4–S37: MS and NMR spectral data of compounds **1**–**3**.
