# Supplementary material for: Metabolites from Streptomyces aureus (VTCC43181) and Their Inhibition of Mycobacterium tuberculosis ClpC1 Protein
Source: Molecules. 2024 Feb 4;29(3):720. doi: 10.3390/molecules29030720 (PMC10856564; doi:10.3390/molecules29030720)
Supplement: Supplementary file 1 [file molecules-29-00720-s001.zip › molecules-2814652-supplementary.pdf]

METABOLITES FROM *STREPTOMYCES AUREUS* (VTCC43181) AND THEIR INHIBITION OF *MYCOBACTERIUM TUBERCULOSIS* CLPC1 PROTEIN

Thao Thi Phuong Tran [1,2,*], Ni Ngoc Thi Huynh [2,3], Ninh Thi Pham [1], Dung Thi Nguyen [1], Chien Van Tran [1], Uyen Quynh Nguyen [4], Anh Ngoc Ho [5], Joo-Won Suh [6], Jinhua Cheng [6], Thao Kim Nu Nguyen [7], Sung Van Tran [1,2] and Duc Minh Nguyen [8,*]

[1]*Institute of Chemistry, Vietnam Academy of Science and Technology (VAST), 18 Hoang Quoc Viet Road, Cau Giay, Hanoi 10000, Vietnam*
[2]*Faculty of chemistry, Graduate University of Science and Technology, VAST, 18 Hoang Quoc Viet Road, Cau Giay, Hanoi 10000, Vietnam*
[3] *Faculty of Natural Sciences, Phu Yen University, 01 Nguyen Van Huyen Road, Tuy Hoa city, Phu Yen, Vietnam*
[4]*Institute of Microbiology and Biotechnology, Vietnam National University, Hanoi*
[5]*Institute of Biotechnology, Vietnam Academy of Science and Technology (VAST), 18 Hoang Quoc Viet Road, Cau Giay, Hanoi 10000, Vietnam*
[6]*Center for Nutraceutical and Pharmaceutical Materials, Myongji University, Yongin 17058, Korea*
[7] *Faculty of Biology, University of Science, Vietnam National University, Hanoi, 334 Nguyen Trai Road, Thanh Xuân, Hanoi, Vietnam*
[8]*Institute of Genome Research, Vietnam Academy of Science and Technology (VAST), 18 Hoang Quoc Viet Road, Cau Giay, Hanoi 10000, Vietnam*

* Corresponding author:
Thao Thi Phuong Tran, Assoc. Prof.
Institute of Chemistry, Vietnam Academy of Science and Technology (VAST), 18 Hoang Quoc Viet Road, Cau Giay, Hanoi, Viet Nam
(E-mail: ntuelam2010@gmail.com)
Duc Minh Nguyen, Dr.
Institute of Genome Research, Vietnam Academy of Science and Technology (VAST), 18 Hoang Quoc Viet Road, Cau Giay, Hanoi 10000, Viet Nam
(E-mail: nmduc@igr.ac.vn)

SUPPLEMENTARY DATA

Figure S1. 16S rDNA sequence of the strain *Streptomyces aureus* (VTCC43181) 2
Figure S2. The phylogenetic tree of the strain *Streptomyces aureus* (VTCC43181) 3
Figure S3. The structures, HMBC and COSY correlations of the isolated compounds (**1-3**) from *Streptomyces aureus* VTCC43181...3
Figure S4. HR-ESI-MS Spectroscopic Data of Compound **1**...4
Figure S5. ^{1}H-NMR Spectrum of Compound **1** in CD_3OD...4
Figure S6. ^{1}H-NMR Spectrum of Compound **1** in CD_3OD (extension) 5
Figure S7. ^{13}C-NMR Spectrum of Compound **1** in CD_3OD..5
Figure S8. ^{13}C-NMR Spectrum of Compound **1** in CD_3OD (extension)..6
Figure S9. DEPT Spectrum of Compound **1** in CD_3OD..6
Figure S10. DEPT Spectrum of Compound **1** in CD_3OD (extension)..7
Figure S11. HSQC Spectrum of Compound **1** in CD_3OD 7
Figure S12. HSQC Spectrum of Compound **1** in CD_3OD (extension) 8
Figure S13. HMBC Spectrum of Compound **1** in CD_3OD 8
Figure S14. HMBC Spectrum of Compound **1** in CD_3OD (extension) 9
Figure S15. HMBC Spectrum of Compound **1** in CD_3OD (extension) 9
Figure S16. COSY Spectrum of Compound **1** in CD_3OD 10
Figure S17. COSY Spectrum of Compound **1** in CD_3OD (extension) 10
Figure S18. (+)-ESI-MS Spectroscopic Data of Compound **2** 11
Figure S19. ^{1}H-NMR Spectrum of Compound **2** in DMSO-d6..11
Figure S20. ^{1}H-NMR Spectrum of Compound **2** in DMSO-d6 (extension)......................................12
Figure S21. ^{13}C-NMR Spectrum of Compound **2** in DMSO-d6...12
Figure S22. ^{13}C-NMR Spectrum of Compound **2** in DMSO-d6 (extension)....................................13
Figure S23. DEPT Spectrum of Compound **2** in DMSO...13
Figure S24. DEPT Spectrum of Compound **2** in DMSO-d6 (extension)...14
Figure S25. HR-ESI-MS Spectroscopic Data of Compound **3**..14
Figure S26. ^{1}H-NMR Spectrum of Compound **3** in CD_3OD 15
Figure S27. ^{1}H-NMR Spectrum of Compound **3** in CD_3OD (extension) 15
Figure S28. ^{1}H-NMR Spectrum of Compound **3** in CD_3OD (extension) 16
Figure S29. ^{13}C-NMR Spectrum of Compound **3** in CD_3OD...16
Figure S30. ^{13}C-NMR Spectrum of Compound **3** in CD_3OD (extension)...17
Figure S31. DEPT Spectrum of Compound **3** in CD_3OD..17
Figure S32. DEPT Spectrum of Compound **3** in CD_3OD (extension)..18
Figure S33. HSQC Spectrum of Compound **3** in CD_3OD 18
Figure S34. HSQC Spectrum of Compound **3** in CD_3OD (extension) 19
Figure S35. HMBC Spectrum of Compound **3** in CD_3OD 19
Figure S36. HMBC Spectrum of Compound **3** in CD_3OD (extension)...20
Figure S37. HMBC Spectrum of Compound **3** in CD_3OD (extension)...20

GGCGTGCTTAACACATGCAAGTCGAACGATGAAGCCCTTCGGGGTGGATT
AGTGGCGAACGGGTGAGTAACACGTGGGCAATCTGCCCTGCACTCTGGG
ACAAGCCCTGGAAACGGGGTCTAATACCGGATGACACCCCCTCTCGCATG
GGAGGGGGTTGAAAGCTCCGGCGGTGCAGGATGAGCCCGCGGCCTATCA
GCTTGTTGGTGAGGTAGAAGCTCACCAAGGCGACGACGGGTAGCCGGCC
TGAGAGGGCGACCGGCCACACTGGGACTGAGACACGGCCCAGACTCCTA
CGGGAGGCAGCAGTGGGGAATATTGCACAATGGGCGAAAGCCTGATGCA
GCGACGCCGCGTGAGGGATGACGGCCTTCGGGTTGTAAACCTCTTTCAGC
AGGGAAGAAGCGAAAGTGACGGTACCTGCAGAAGAAGCGCCGGCTAACT
ACGTGCCAGCAGCCGCGGTAATACGTAGGGCGCAAGCGTTGTCCGGAATT
ATTGGGCGTAAAGAGCTCGTAGGCGGCTTGTCACGTCGGTTGTGAAAGCC
CGGGGCTTAACCCCGGGTCTGCAGTCGATACGGGCAGGCTAGAGTGTGGT
AGGGGAGATCGGAATTCCTGGTGTAGCGGTGAAATGCGCAGATATCAGG
AGGAACACCGGTGGCGAAGGCGGATCTCTGGGCCATTACTGACGCTGAG
GAGCGAAAGCGTGGGGAGCGAACAGGATTAGATACCCTGGTAGTCCACG
CCGTAAACGGTGGGAACTAGGTGTTGGCGACATTCCACGTCGTCGGTGCC
GCAGCTAACGCATTAAGTTCCCCGCCTGGGGAGTACGGCCGCAAGGCTAA
AACTCAAAGGAATTGACGGGGGCCCGCACAAGCAGCGGAGCATGTGGCT
TAATTCGACGCAACGCGAAGAACCTTACCAAGGCTTGACATATACCGGAA
AGCATCAGAGATGGTGCCCCCCTTGTGGTCGGTATACAGGTGGTGCATGG
CTGTCGTCAGCTCGTGTCGTGAGATGTTGGGTTAAGTCCCGCAACGAGCG
CAACCCTTGTTCTGTGTTGCCAGCATGCCCTTCGGGGTGATGGGGACTCA
CAGGAGACTGCCGGGGTCAACTCGGAGGAAGGTGGGGACGACGTCAAGT
CATCATGCCCCTTATGTCTTGGGCTGCACACGTGCTACAATGGCAGGTAC
AATGAGCTGCGATGCCGCGAGGCGGAGCGAATCTCAAAAAGCCTGTCTC
AGTTCGGATTGGGGTCTGCAACTCGACCCCATGAAGTCGGAGTTGCTAGT
AATCGCAGATCAGCATTGCTGCGGTGAATACGTTCCCGGGCCTTGTACAC
ACCGCCCGTCACGTCACGAAAGTCGGTAACACCCGAAGCCGGTGGCCCA
ACCCCCTTGTGGGGAGGGAGCTGTCGAAGGTGGGACTGGCGATTGGGAC
GAAGTCGTAACAAG

Figure S1. 16S rDNA sequence of the strain *Streptomyces aureus* (VTCC43181)

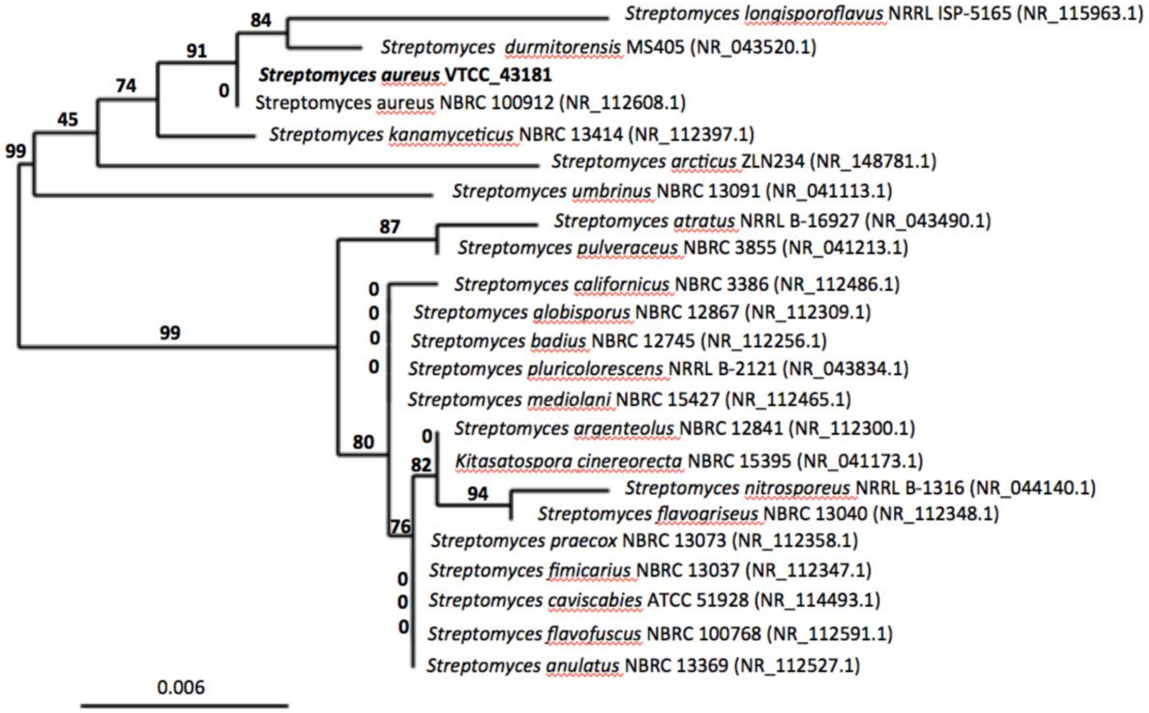

Figure S2. The phylogenetic tree of the strain *Streptomyces aureus* (VTCC43181)

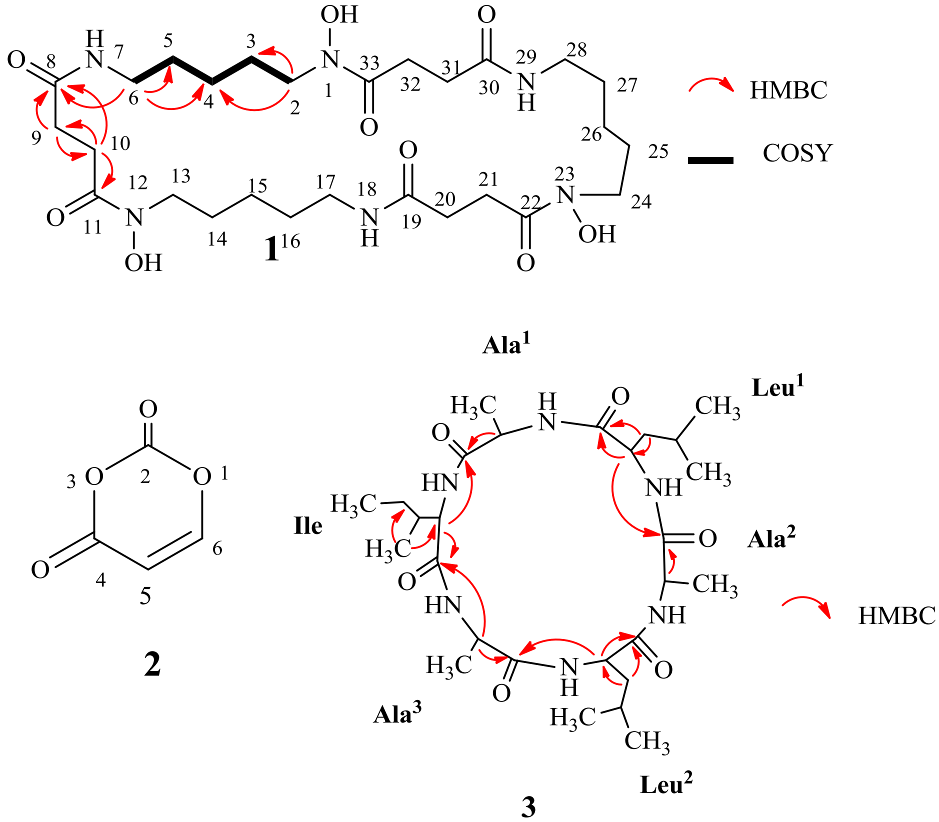

Figure S3. The structures, HMBC and COSY correlations of the isolated compounds (**1-3**) from *Streptomyces aureus* VTCC43181

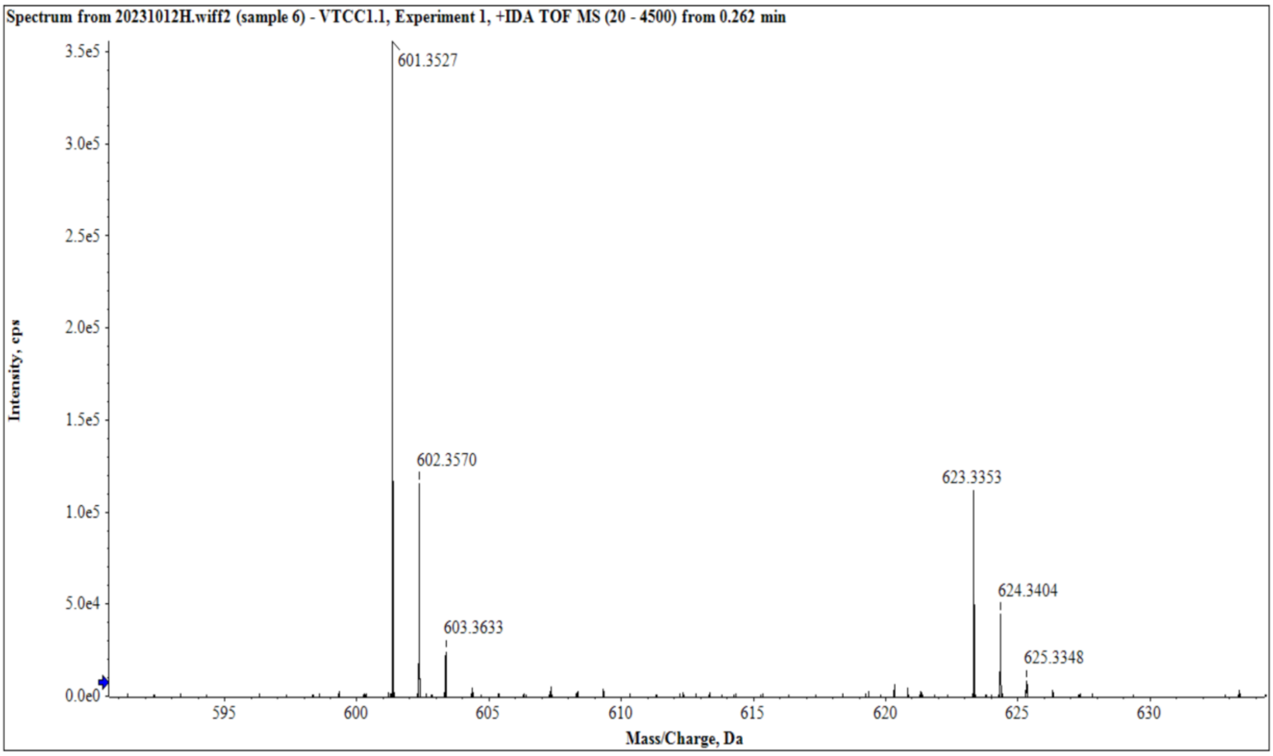

Figure S4. HR-ESI-MS Spectroscopic Data of Compound **1**

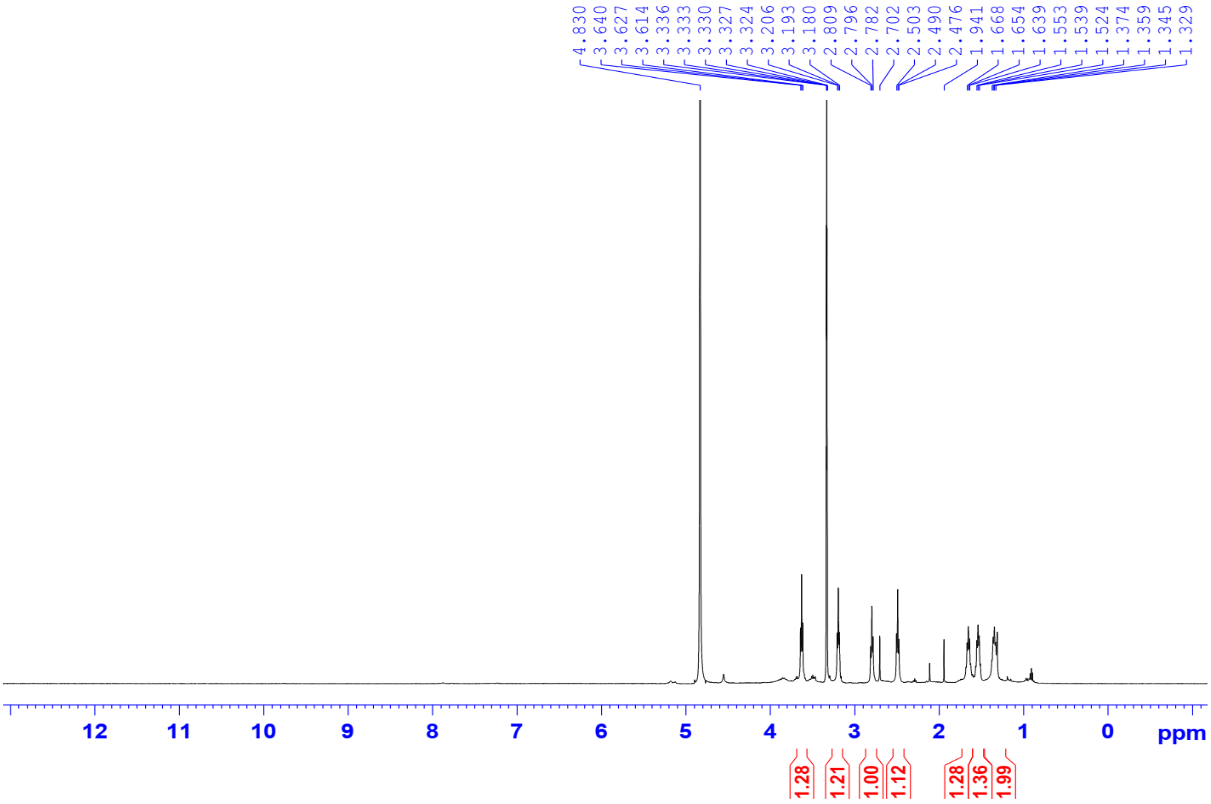

Figure S5. ^{1}H-NMR Spectrum of Compound **1** in CD_3OD

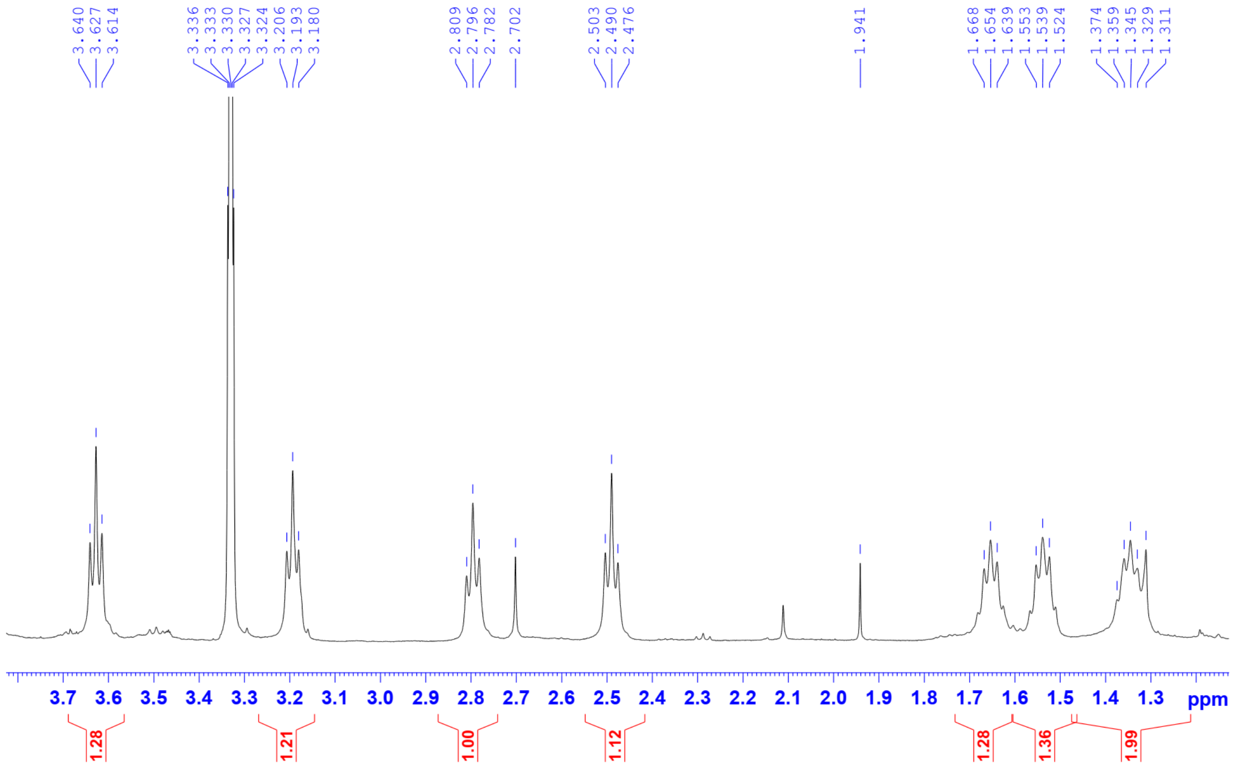

Figure S6. ^{1}H-NMR Spectrum of Compound **1** in CD_3OD (extension)

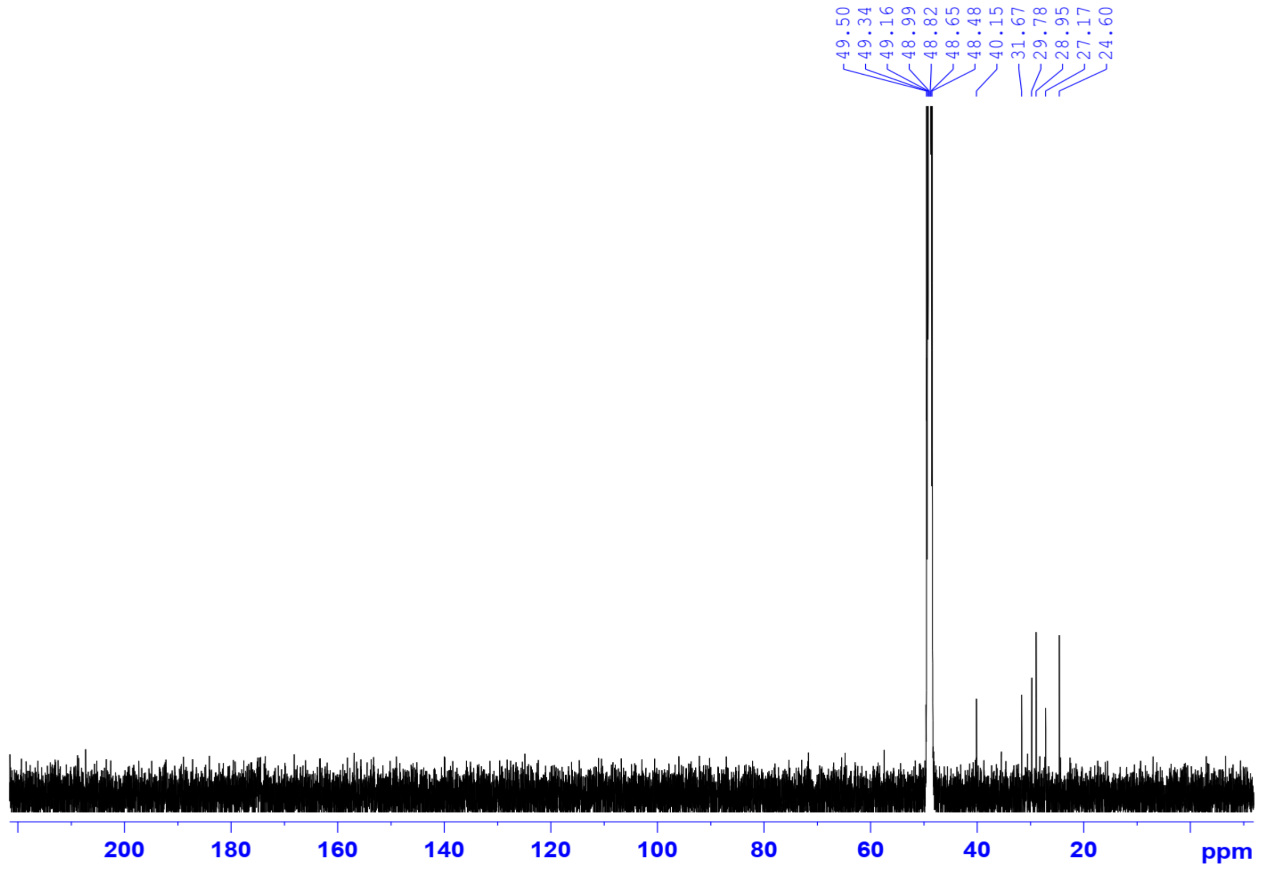

Figure S7. ^{13}C-NMR Spectrum of Compound **1** in CD_3OD

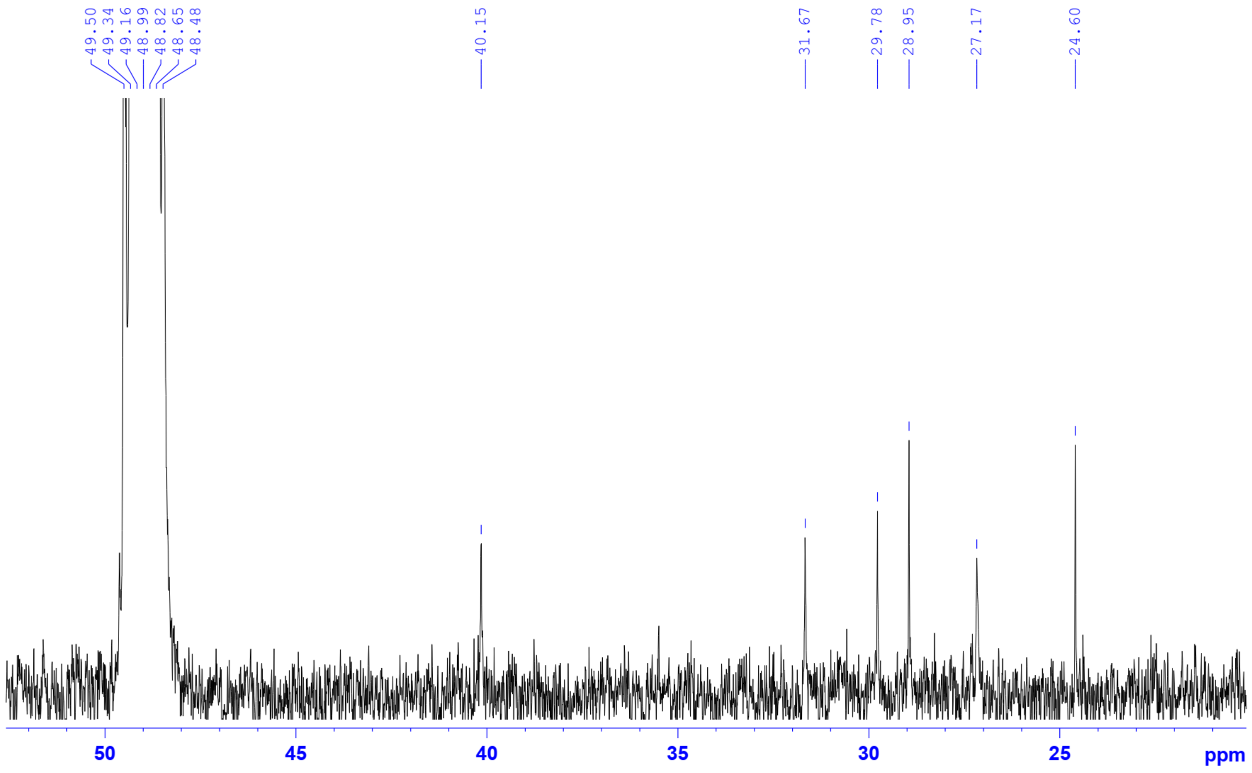

Figure S8. ^{13}C-NMR Spectrum of Compound **1** in CD_3OD (extension)

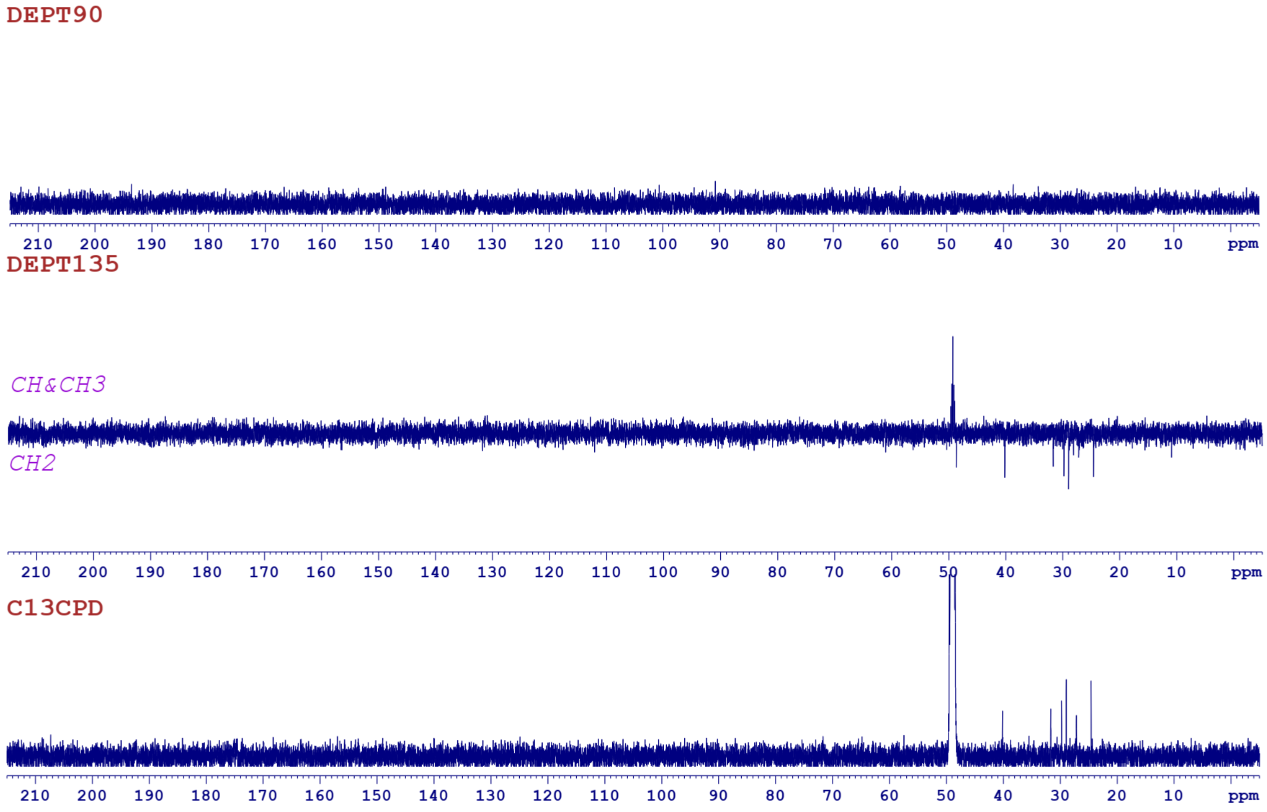

Figure S9. DEPT Spectrum of Compound **1** in CD_3OD

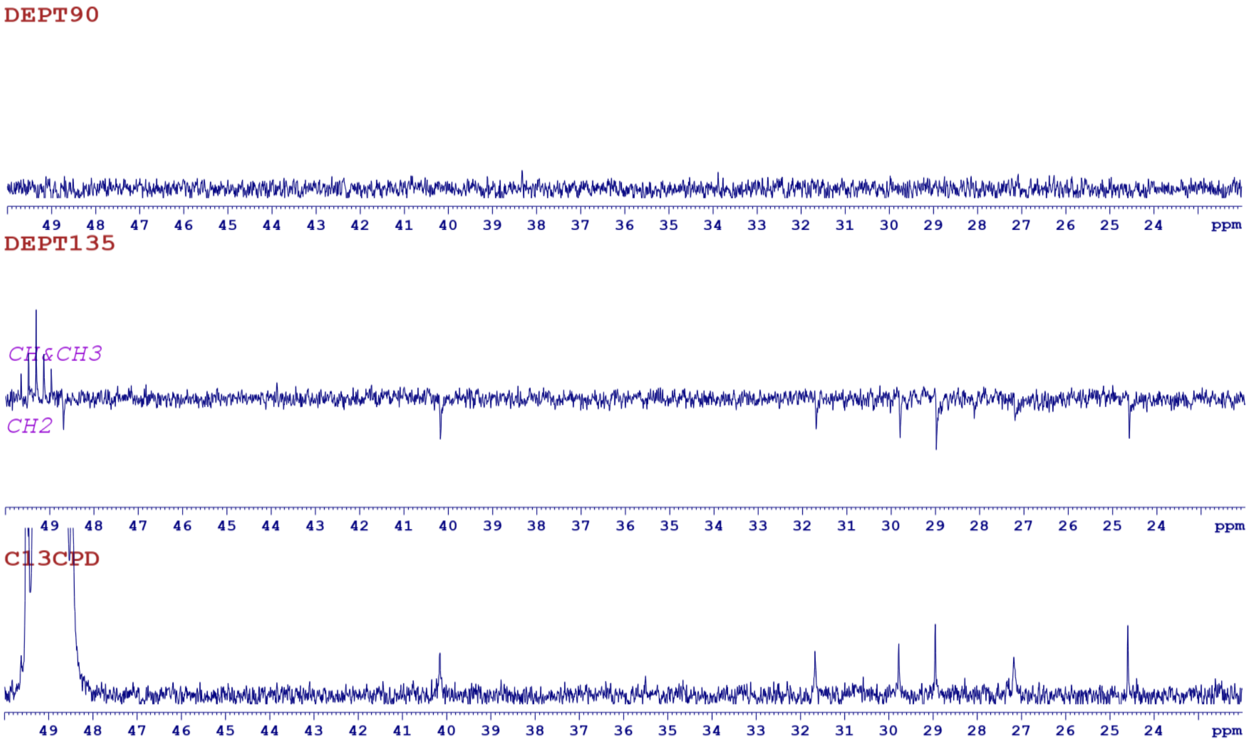

Figure S10. DEPT Spectrum of Compound **1** in CD_3OD (extension)

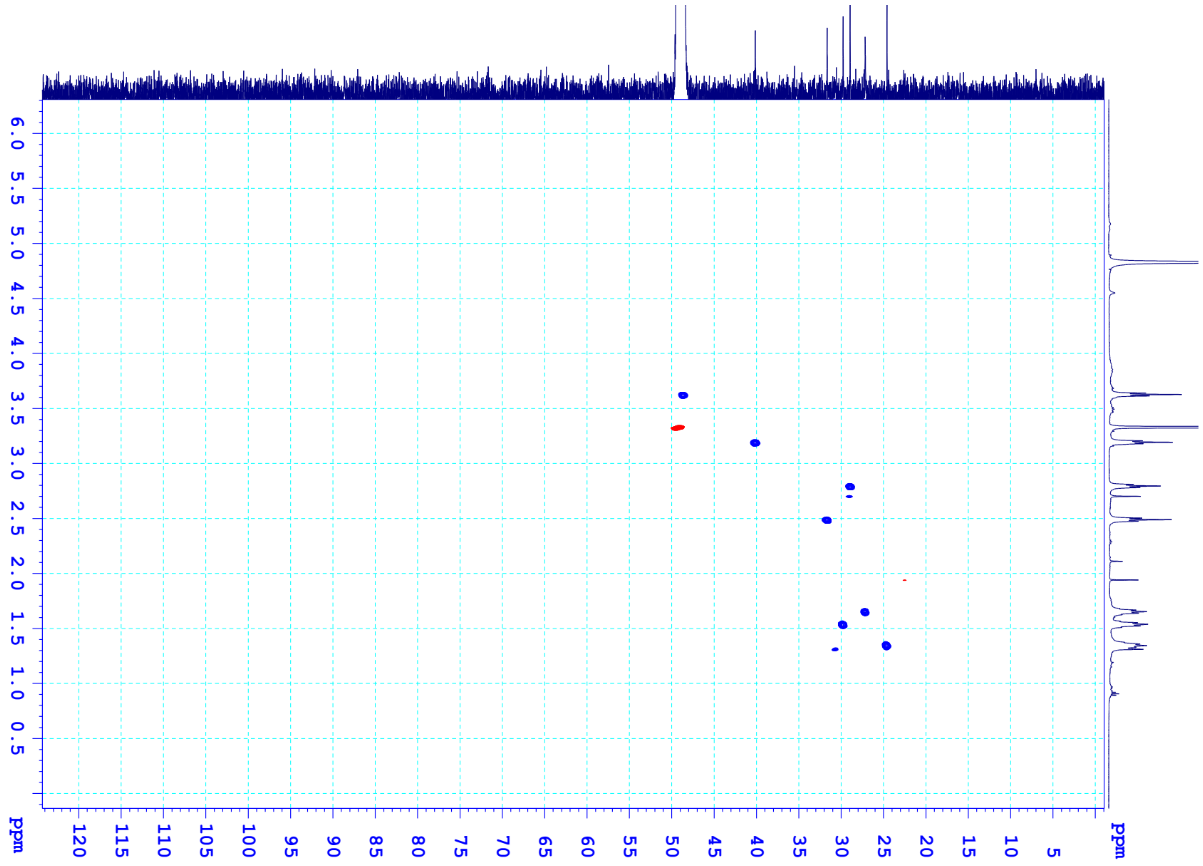

Figure S11. HSQC Spectrum of Compound **1** in CD_3OD

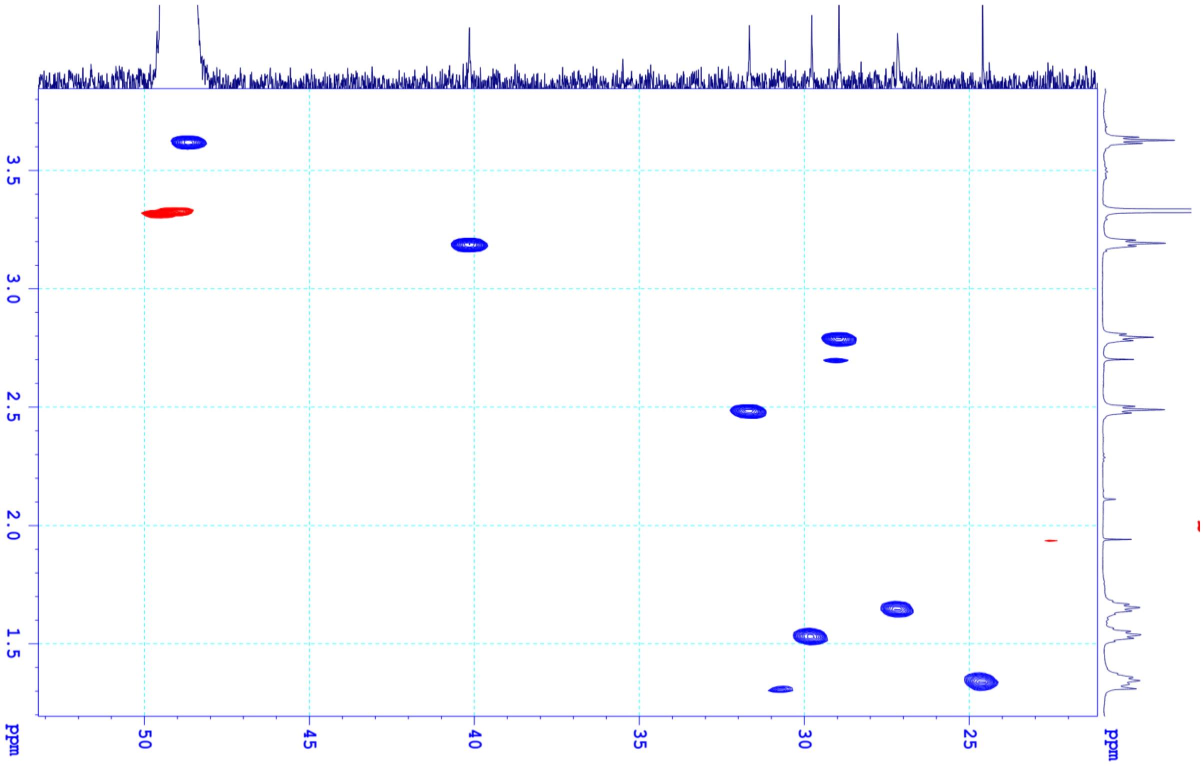

Figure S12. HSQC Spectrum of Compound **1** in CD_3OD (extension)

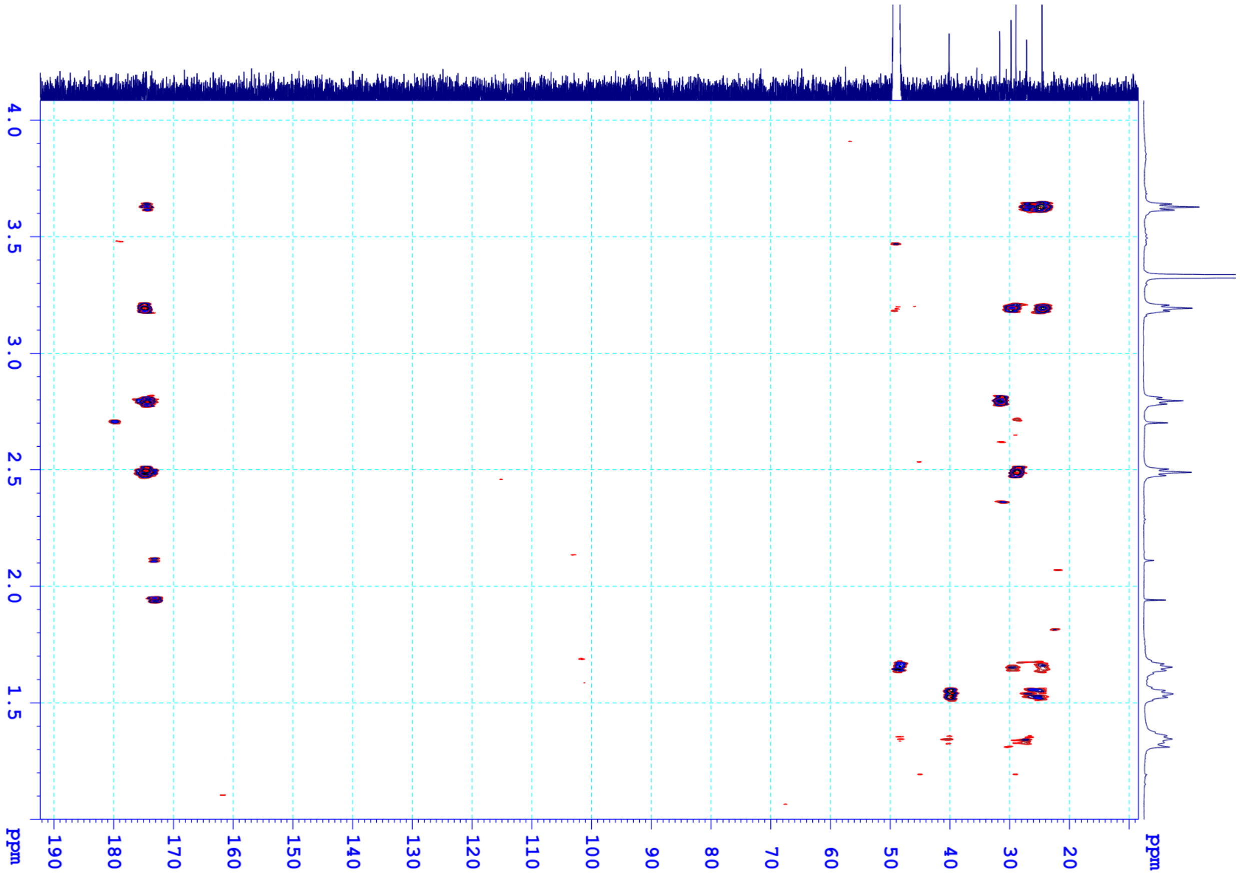

Figure S13. HMBC Spectrum of Compound **1** in CD_3OD

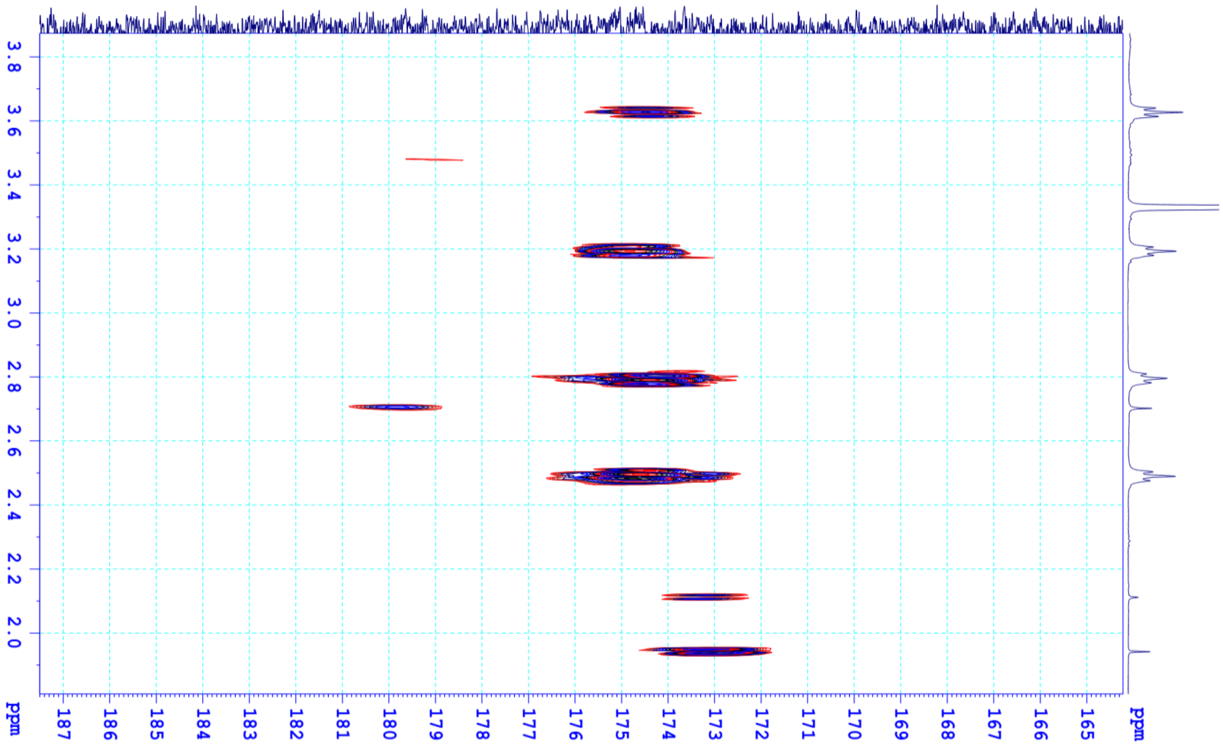

Figure S14. HMBC Spectrum of Compound **1** in CD_3OD (extension)

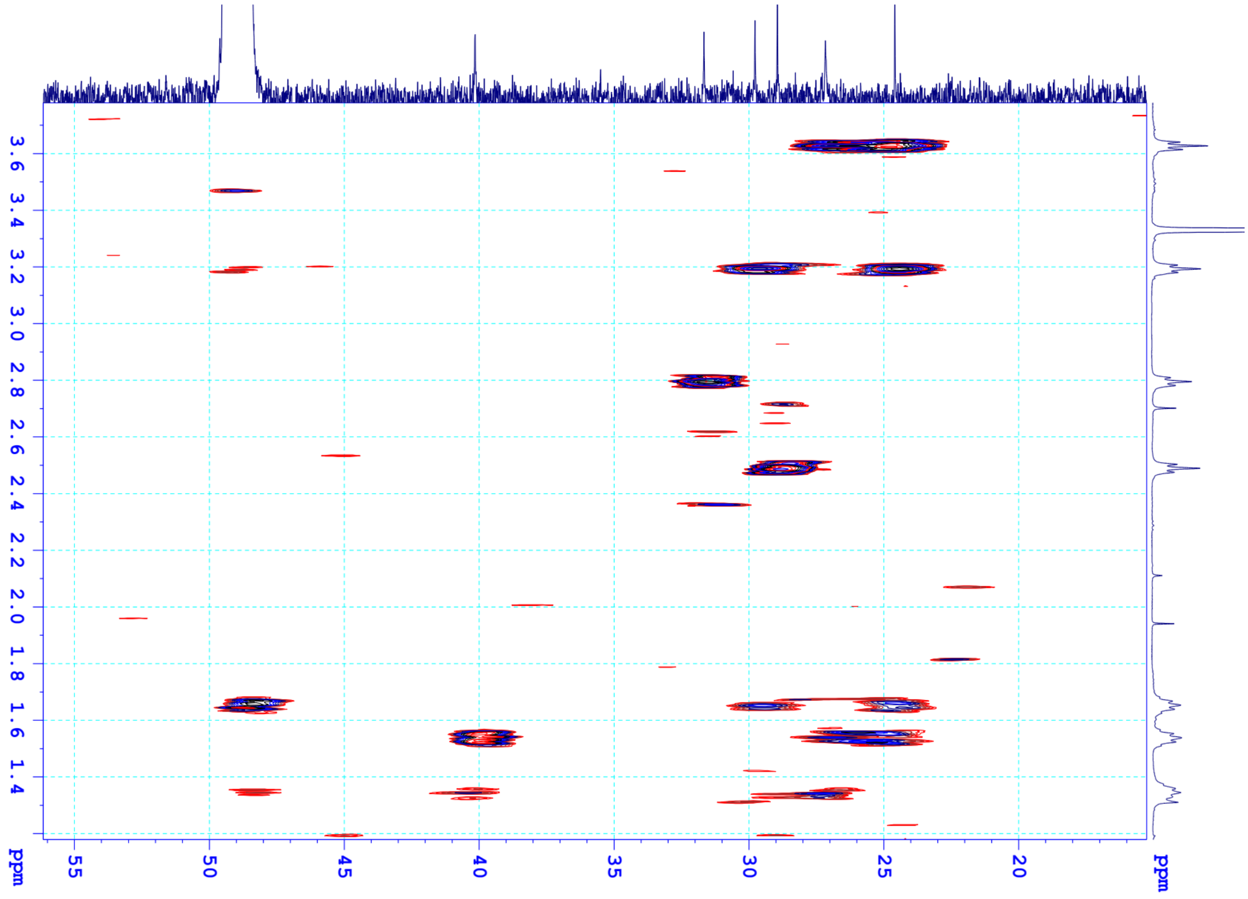

Figure S15. HMBC Spectrum of Compound **1** in CD_3OD (extension)

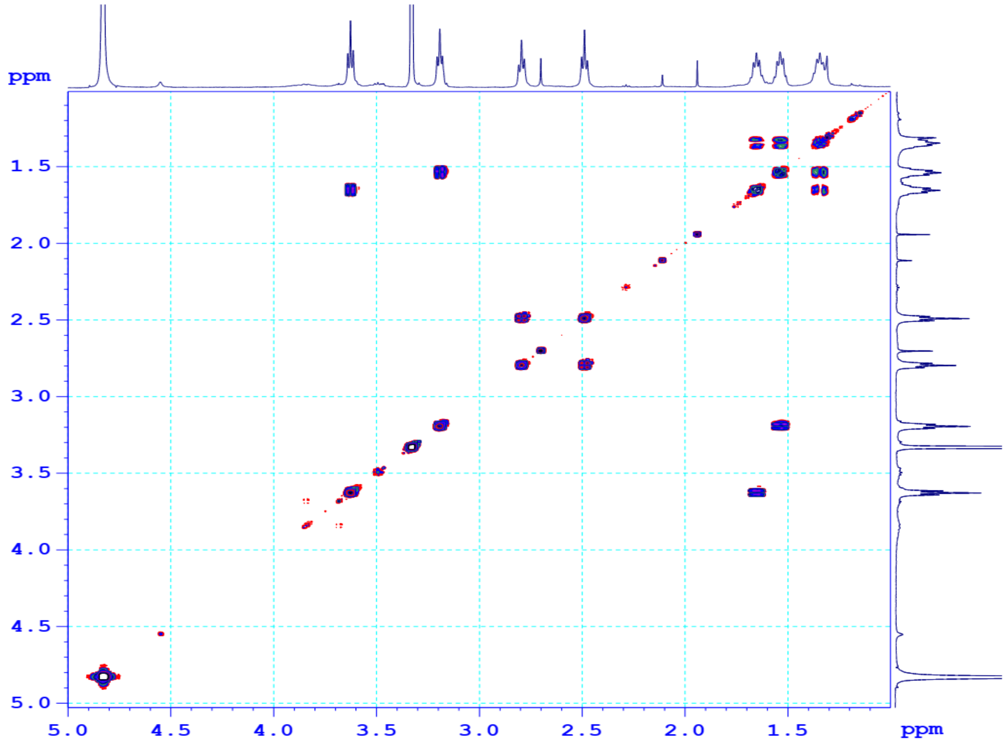

Figure S16. COSY Spectrum of Compound **1** in CD_3OD

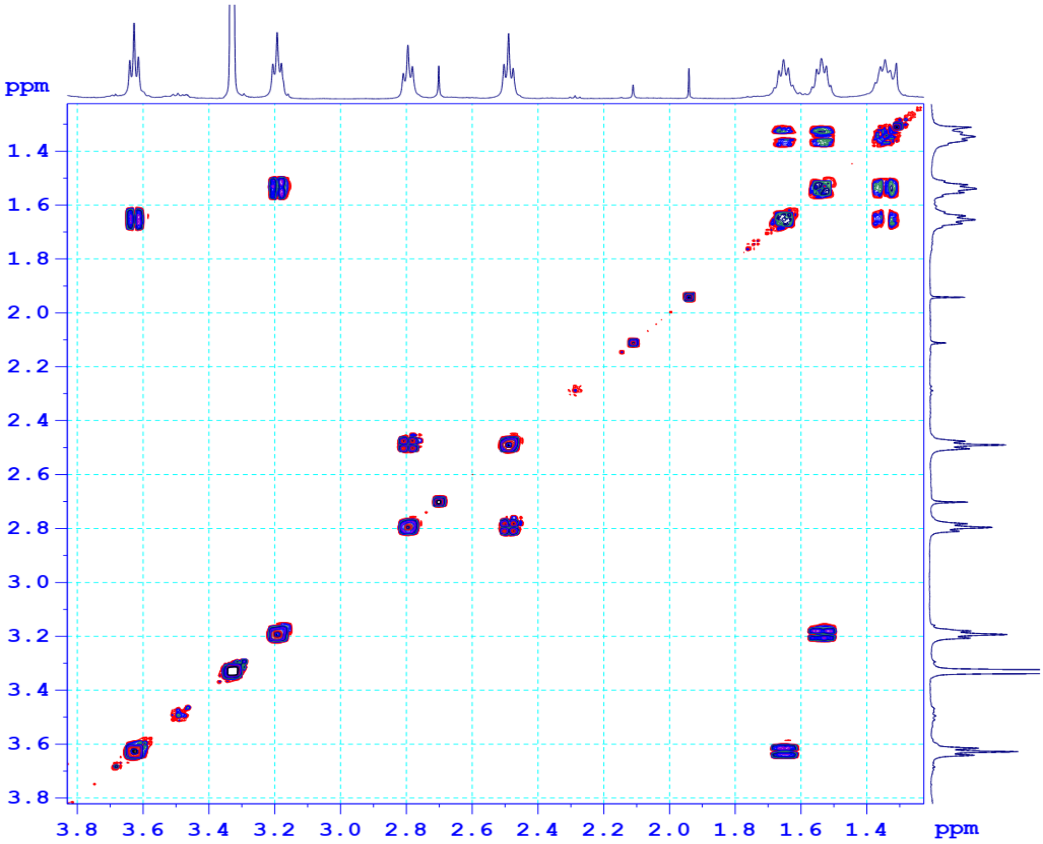

Figure S17. COSY Spectrum of Compound **1** in CD_3OD (extension)

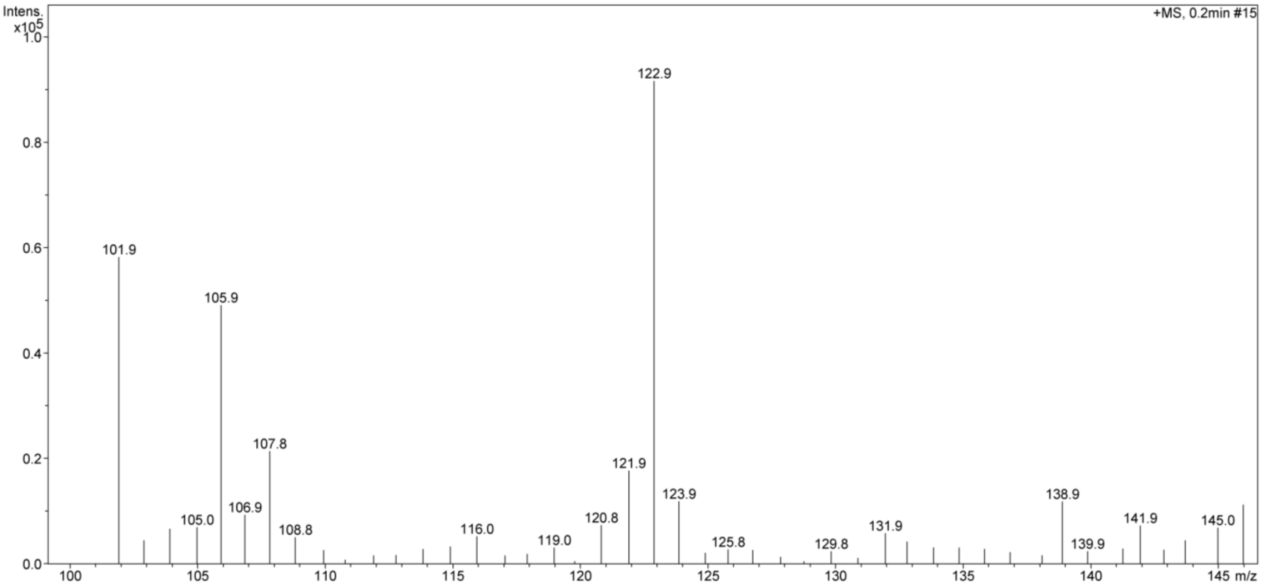

Figure S18. (+)-ESI-MS Spectroscopic Data of Compound **2**

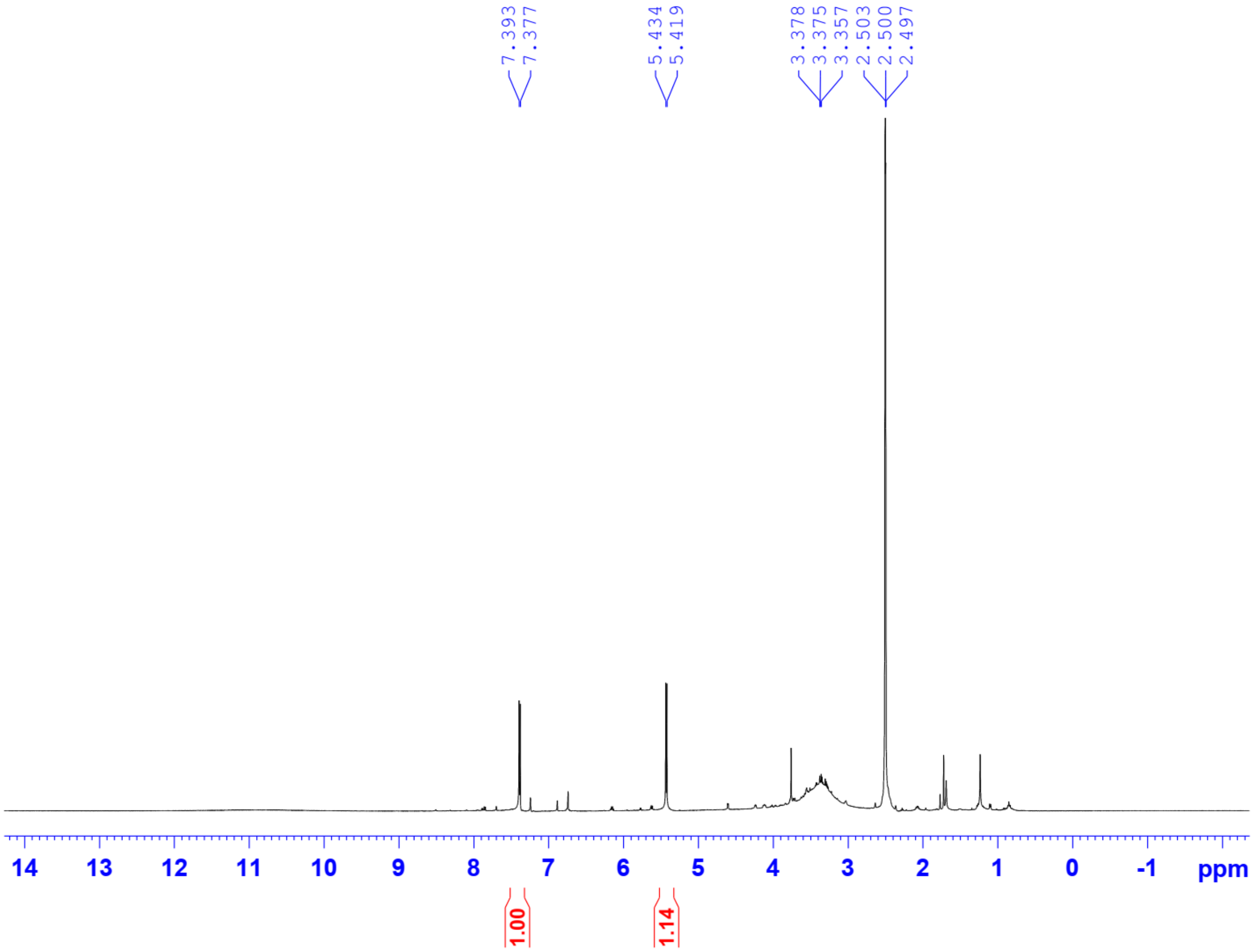

Figure S19. ^{1}H-NMR Spectrum of Compound **2** in DMSO-d6

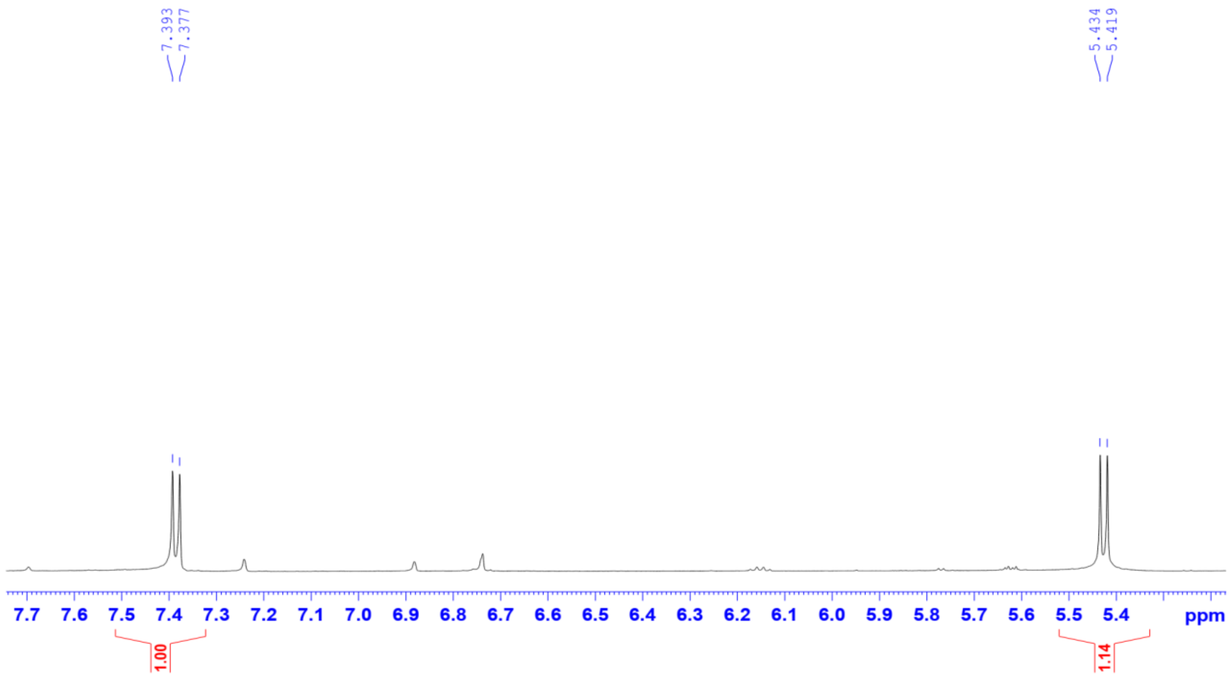

Figure S20. ^{1}H-NMR Spectrum of Compound **2** in DMSO-d6 (extension)

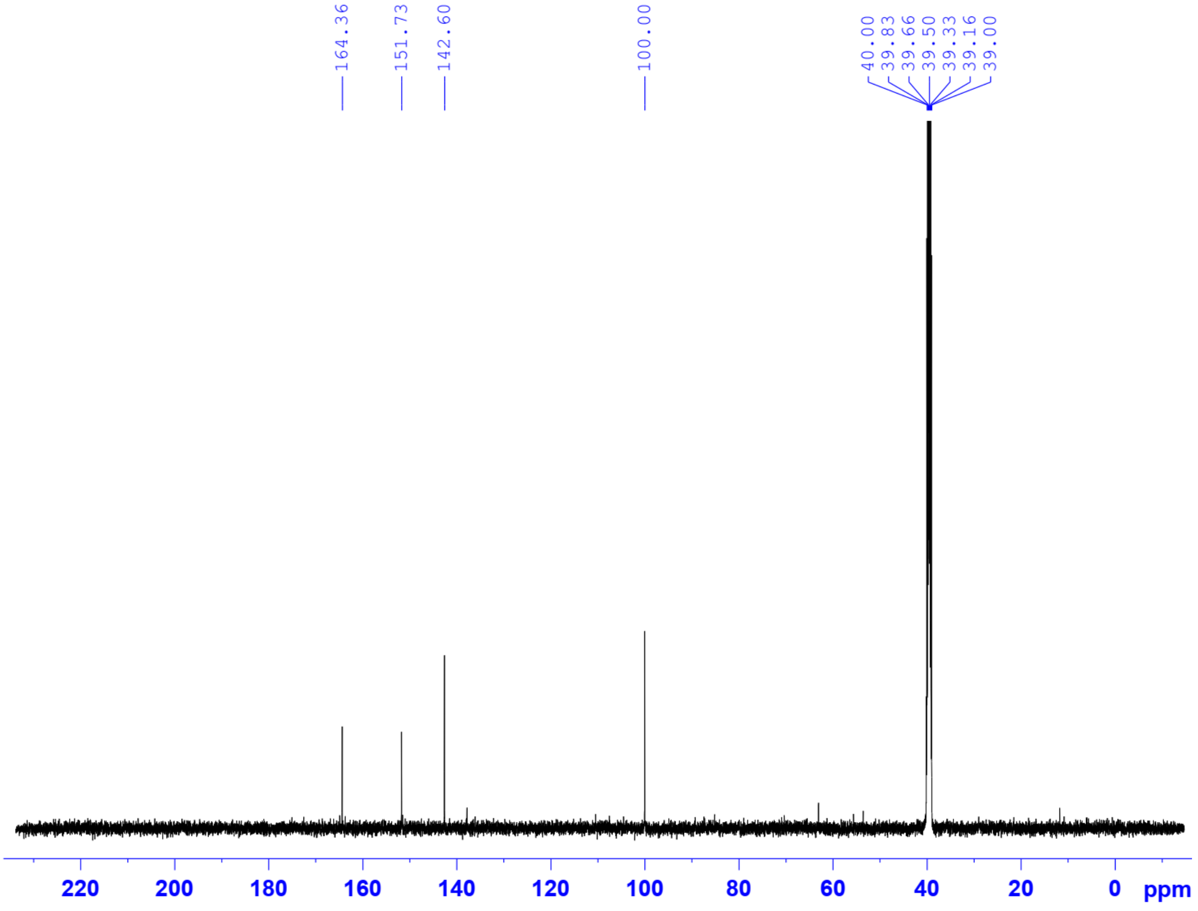

Figure S21. ^{13}C-NMR Spectrum of Compound **2** in DMSO-d6

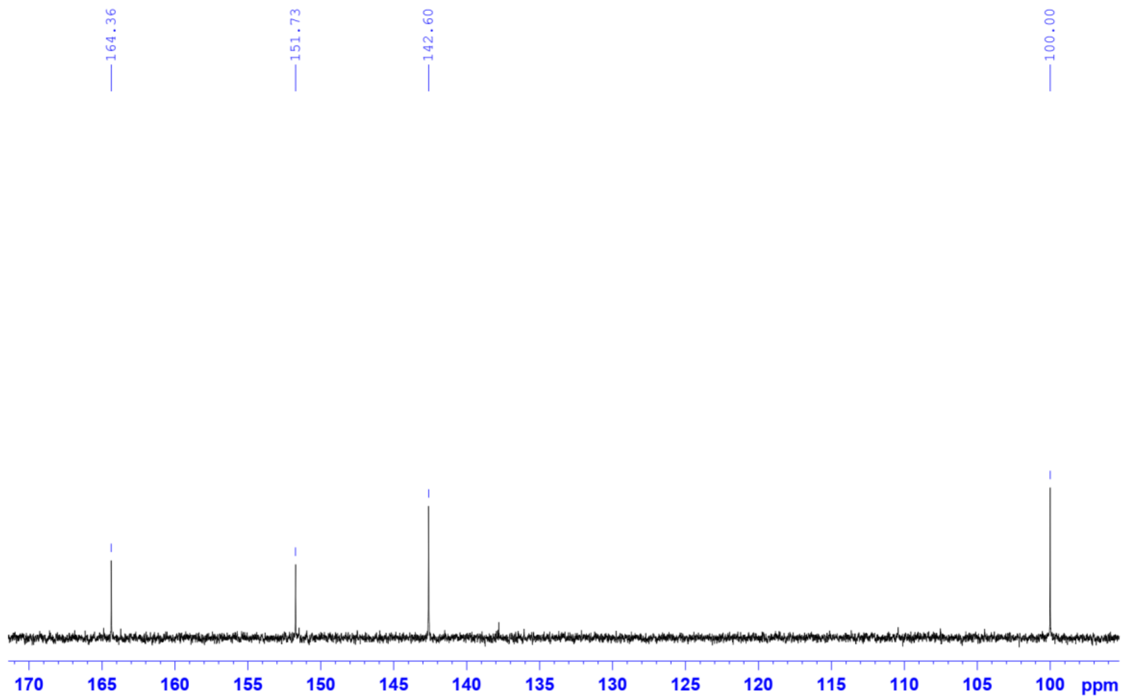

Figure S22. ^{13}C-NMR Spectrum of Compound **2** in DMSO-d6 (extension)

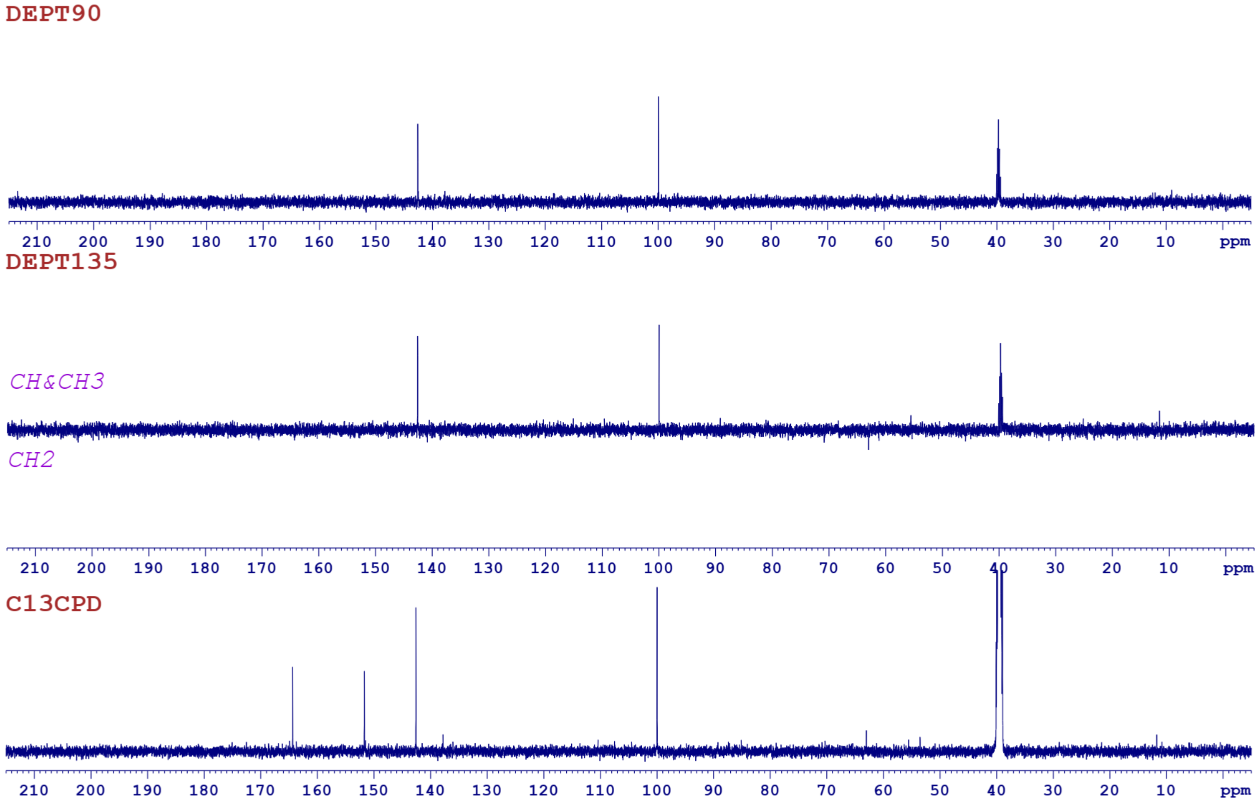

Figure S23. DEPT Spectrum of Compound **2** in DMSO-d6

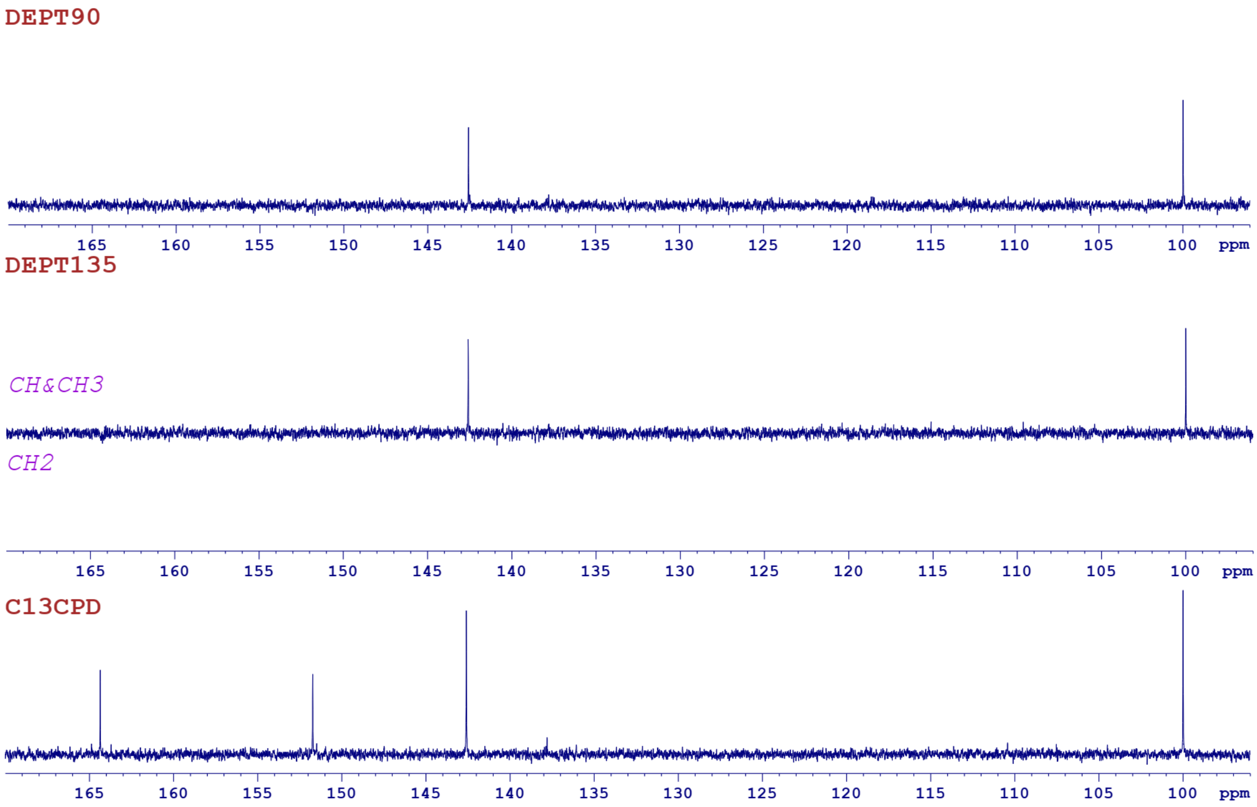

Figure S24. DEPT Spectrum of Compound **2** in DMSO-d6 (extension)

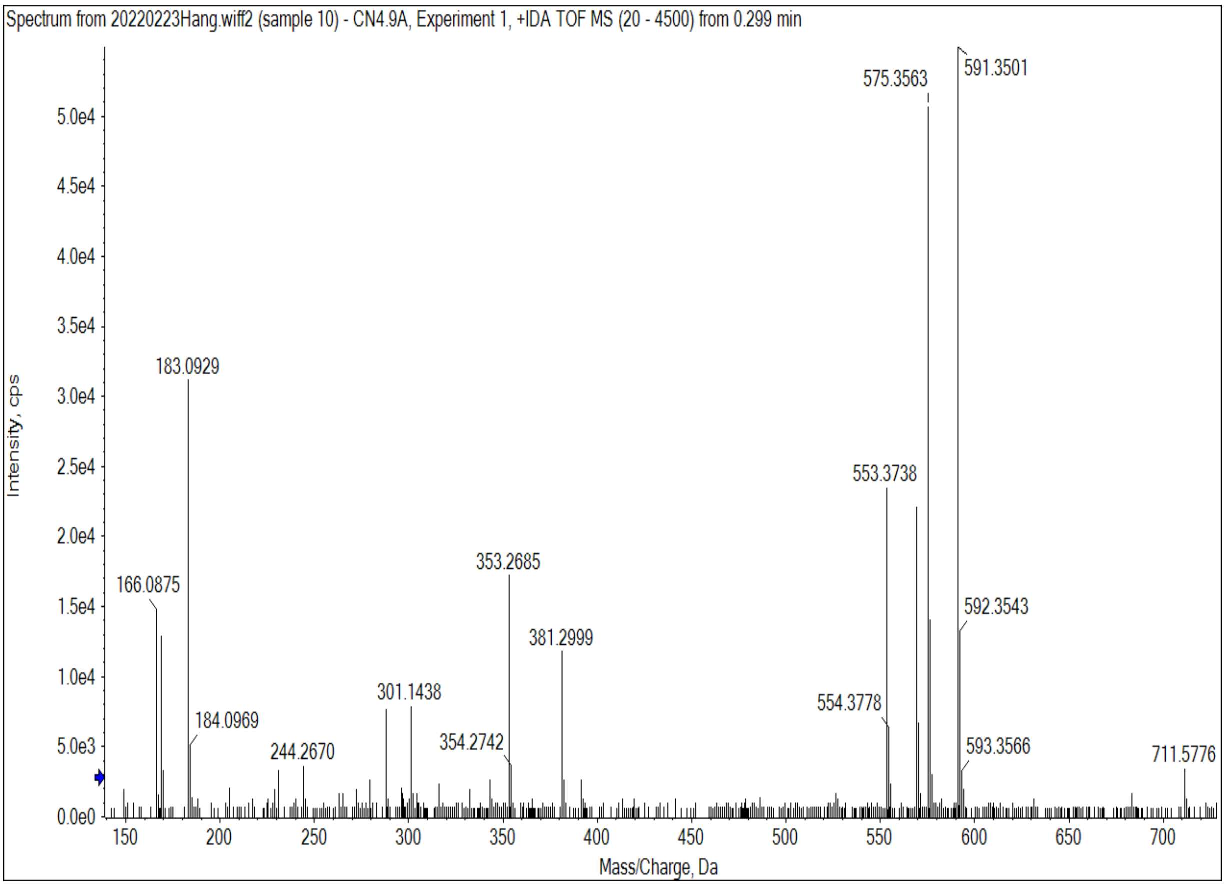

Figure S25. HR-ESI-MS Spectroscopic Data of Compound **3**

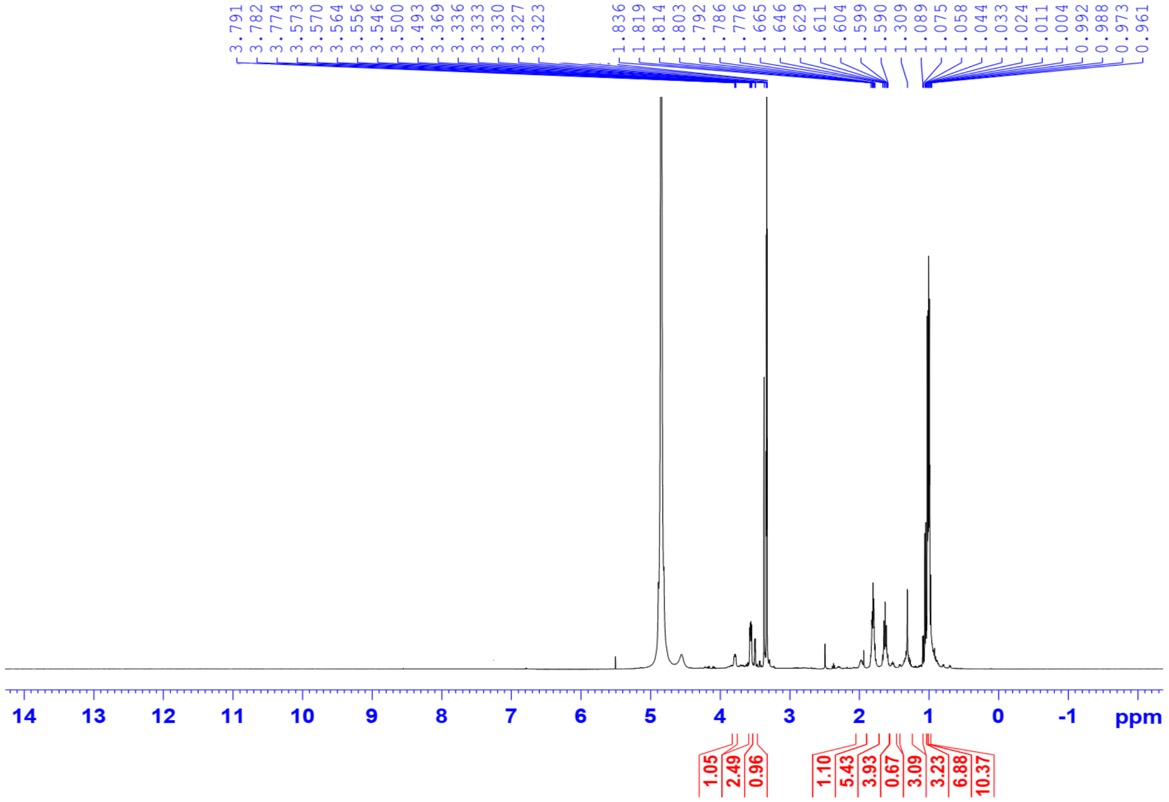

Figure S26. ^{1}H-NMR Spectrum of Compound **3** in CD_3OD

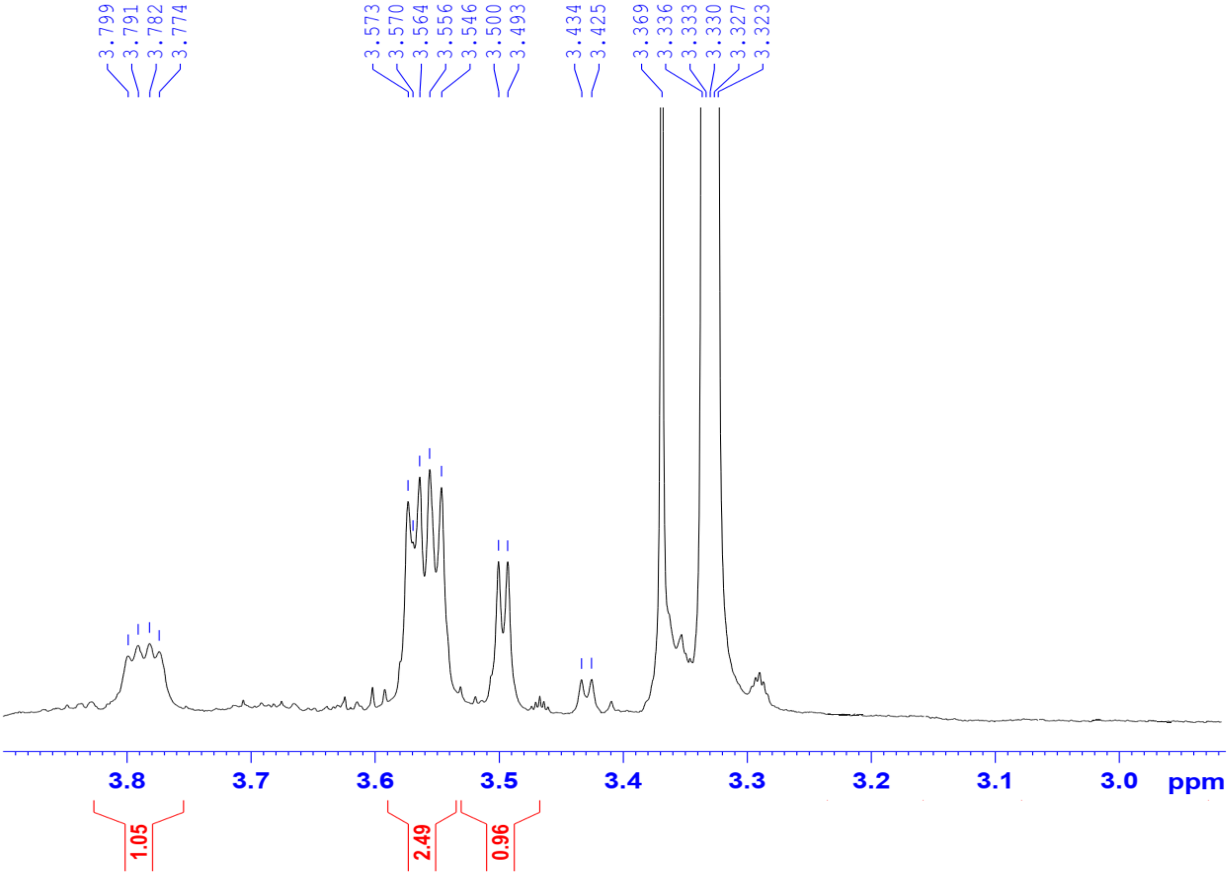

Figure S27. ^{1}H-NMR Spectrum of Compound **3** in CD_3OD (extension)

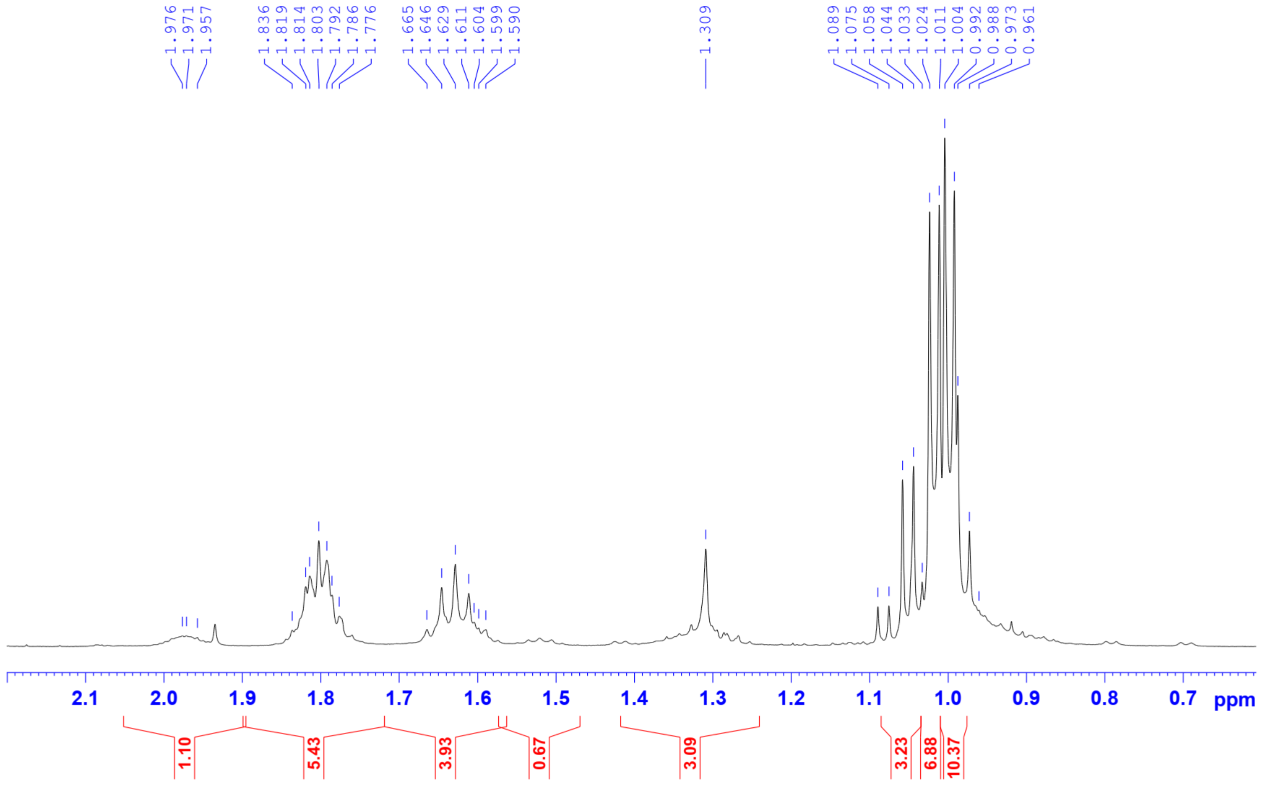

Figure S28. ^{1}H-NMR Spectrum of Compound **3** in CD_3OD (extension)

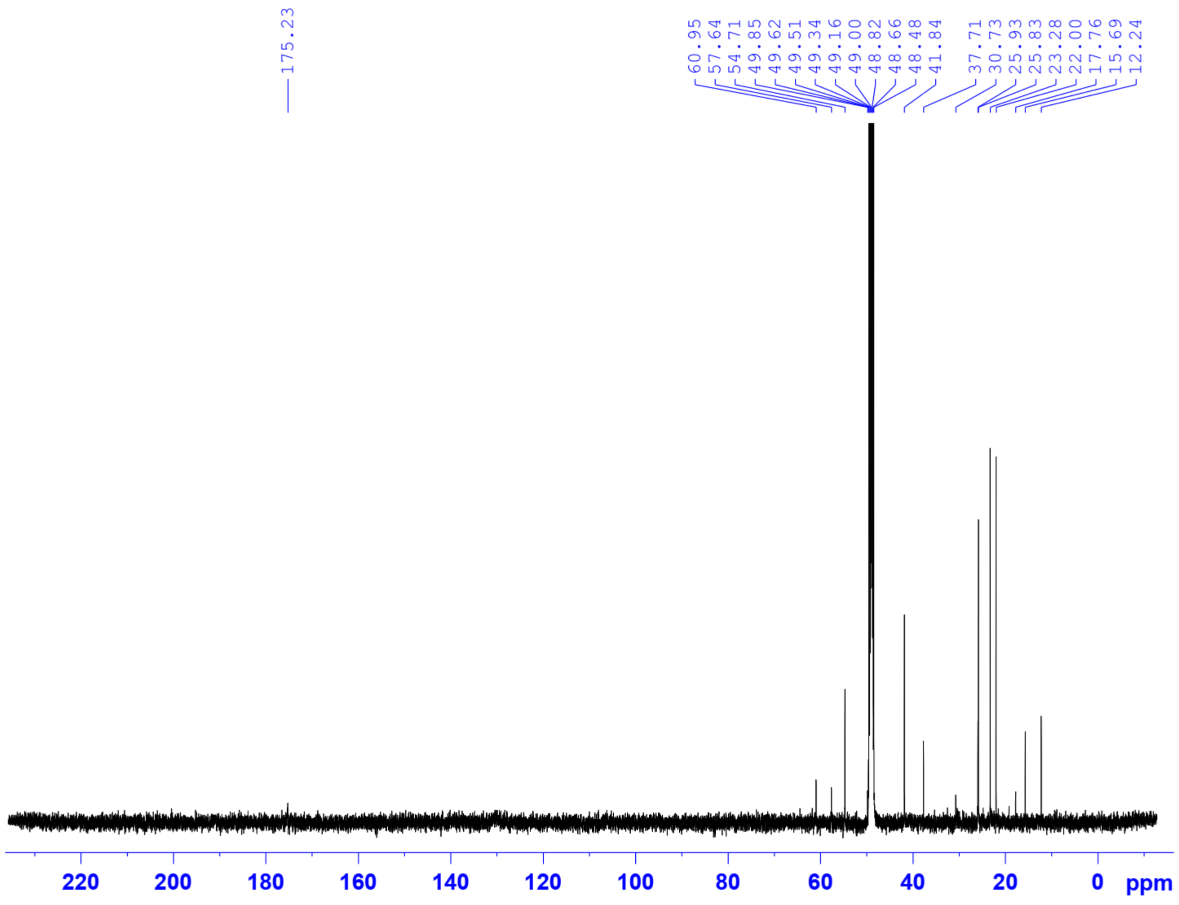

Figure S29. ^{13}C-NMR Spectrum of Compound **3** in CD_3OD

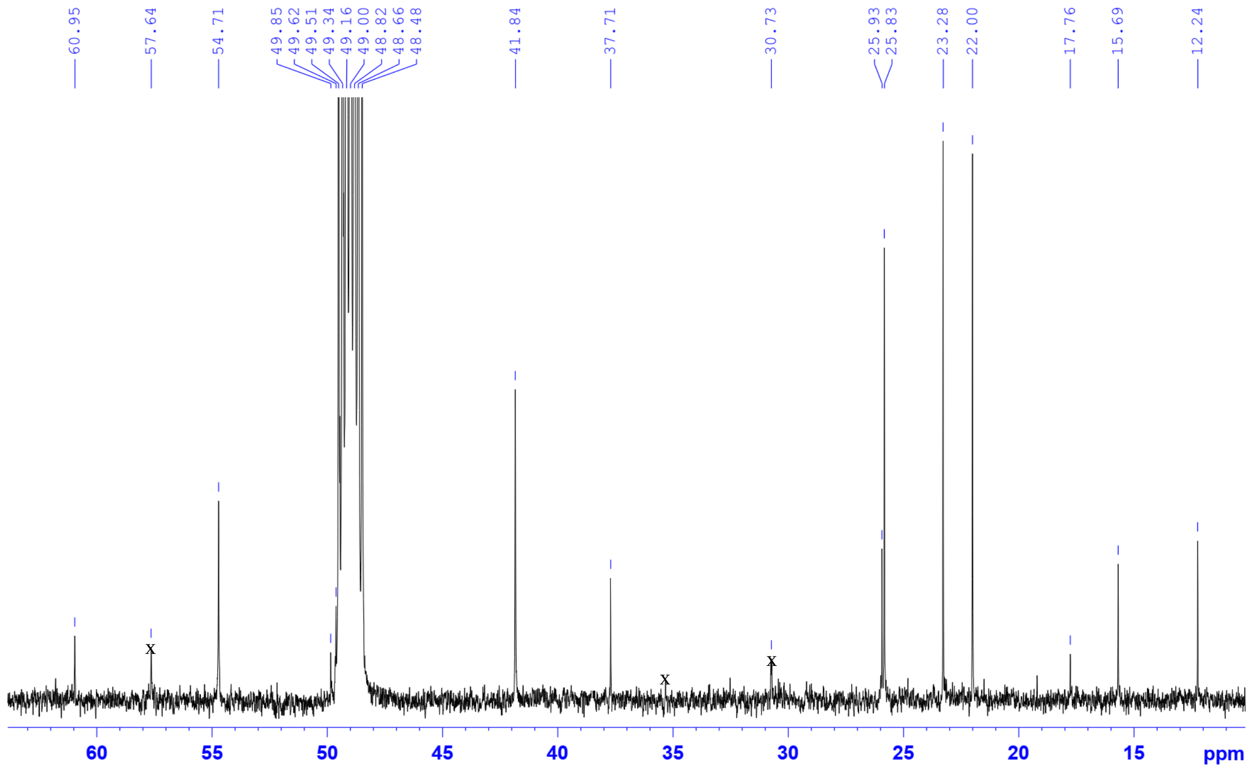

Figure S30. ^{13}C-NMR Spectrum of Compound **3** in CD_3OD (extension)

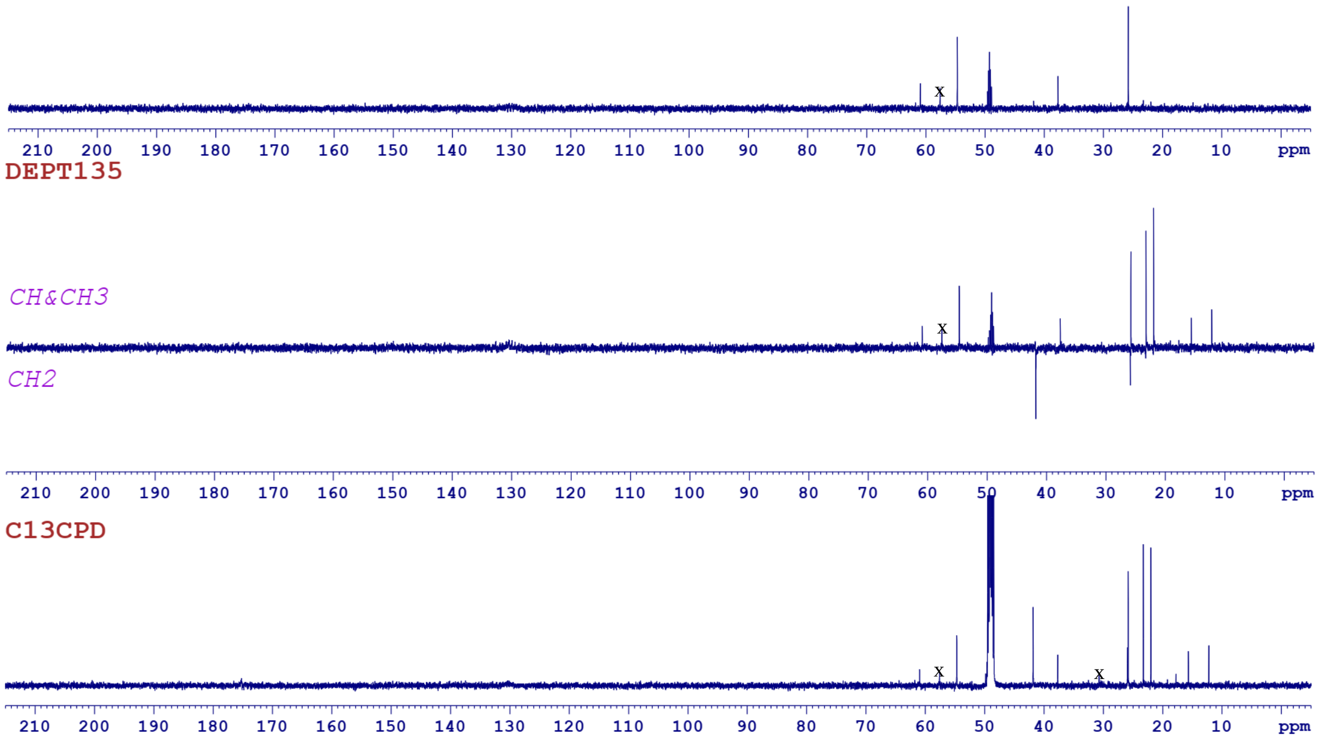

Figure S31. DEPT Spectrum of Compound **3** in CD_3OD

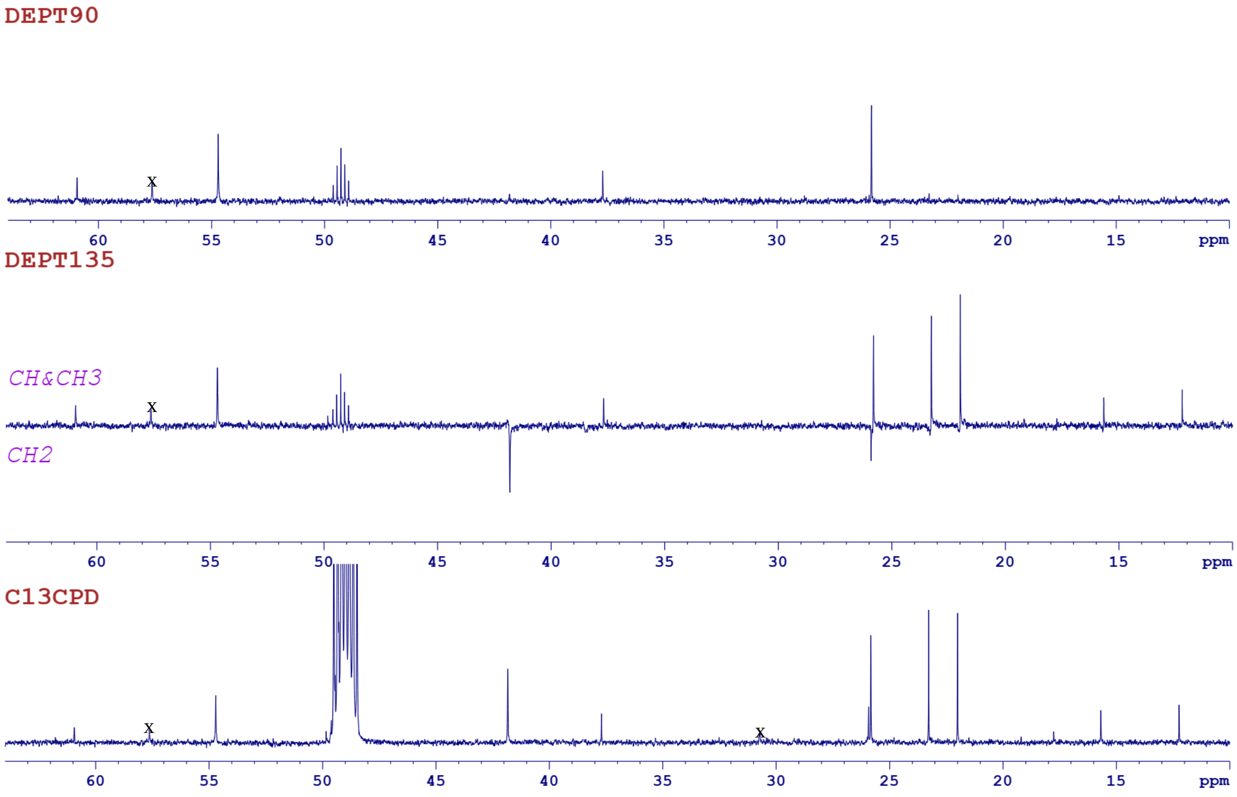

Figure S32. DEPT Spectrum of Compound **3** in CD_3OD (extension)

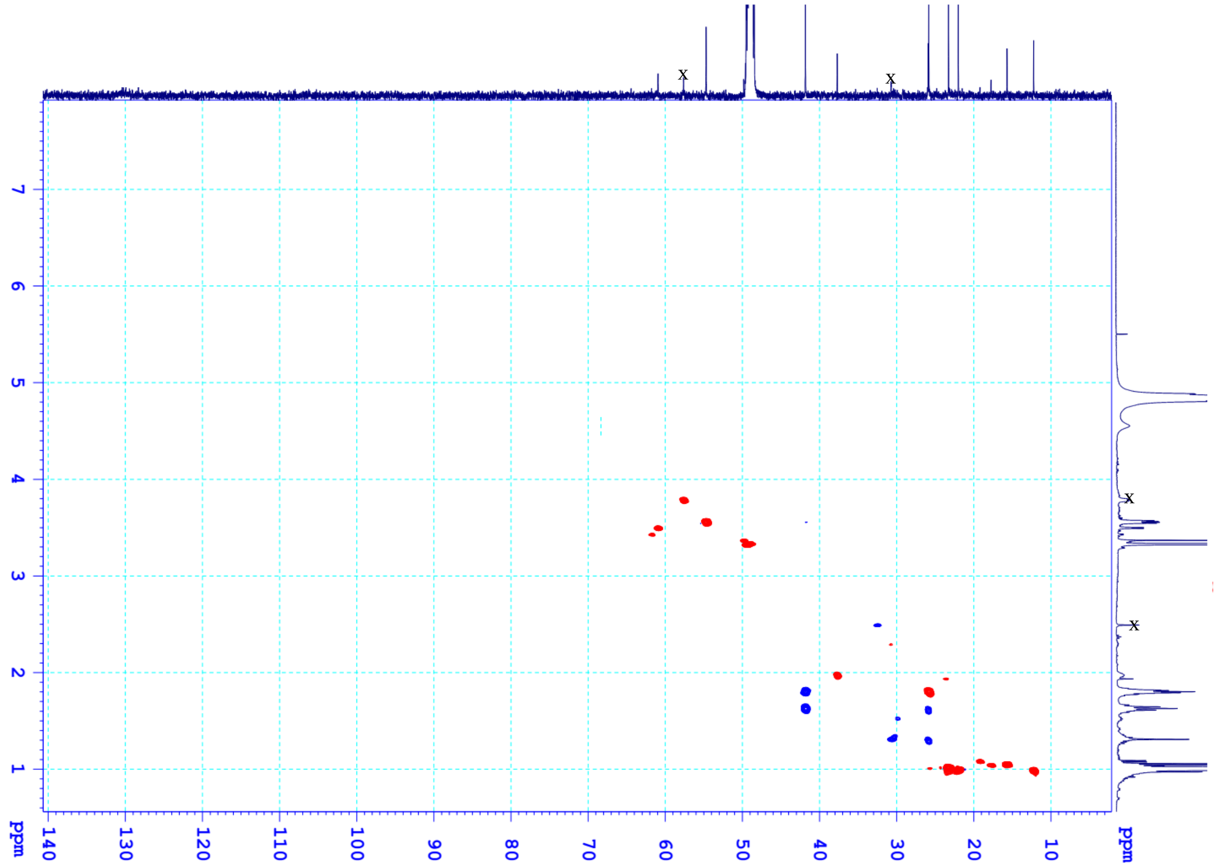

Figure S33. HSQC Spectrum of Compound **3** in CD_3OD

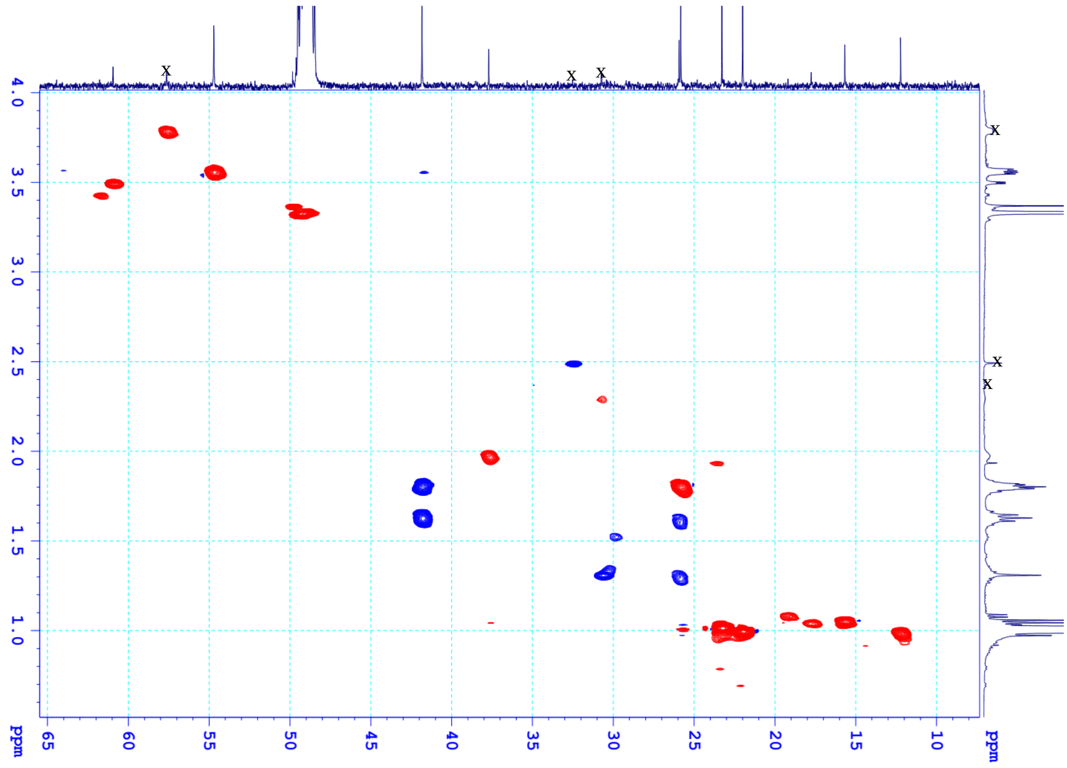

Figure S34. HSQC Spectrum of Compound **3** in CD_3OD (extension)

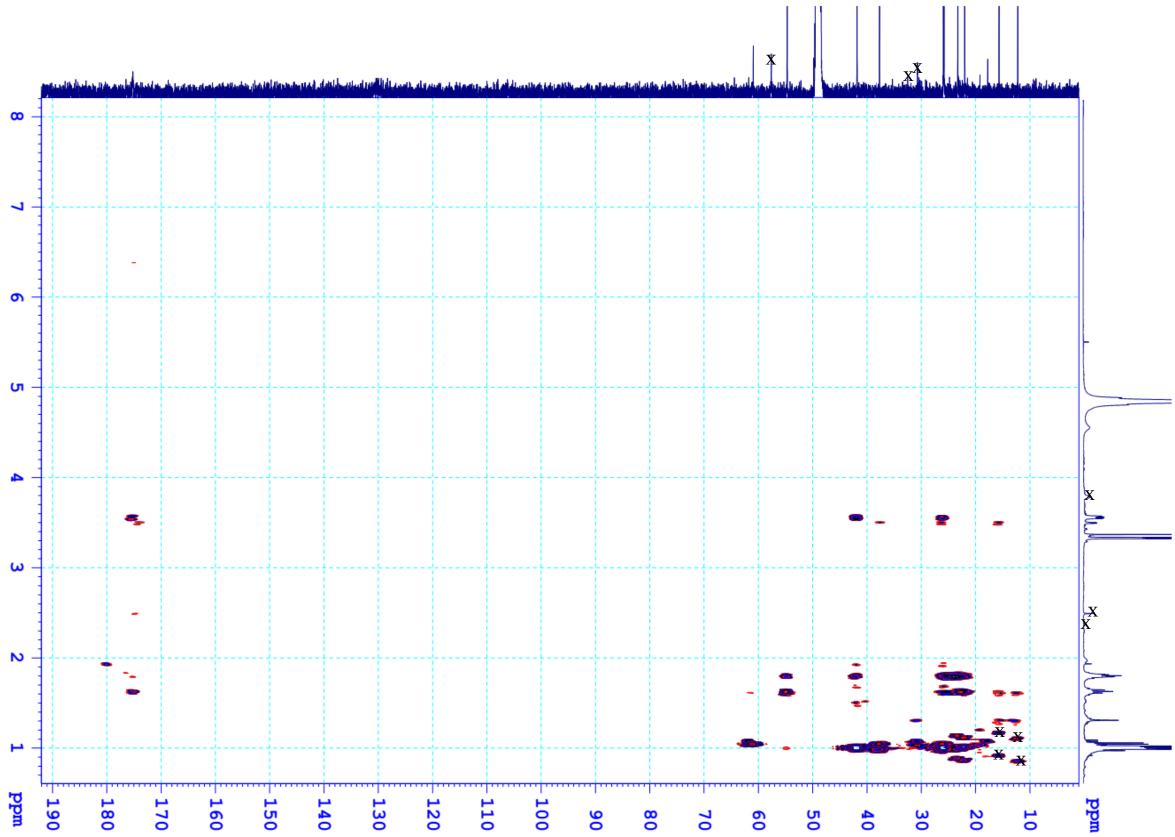

Figure S35. HMBC Spectrum of Compound **3** in CD_3OD

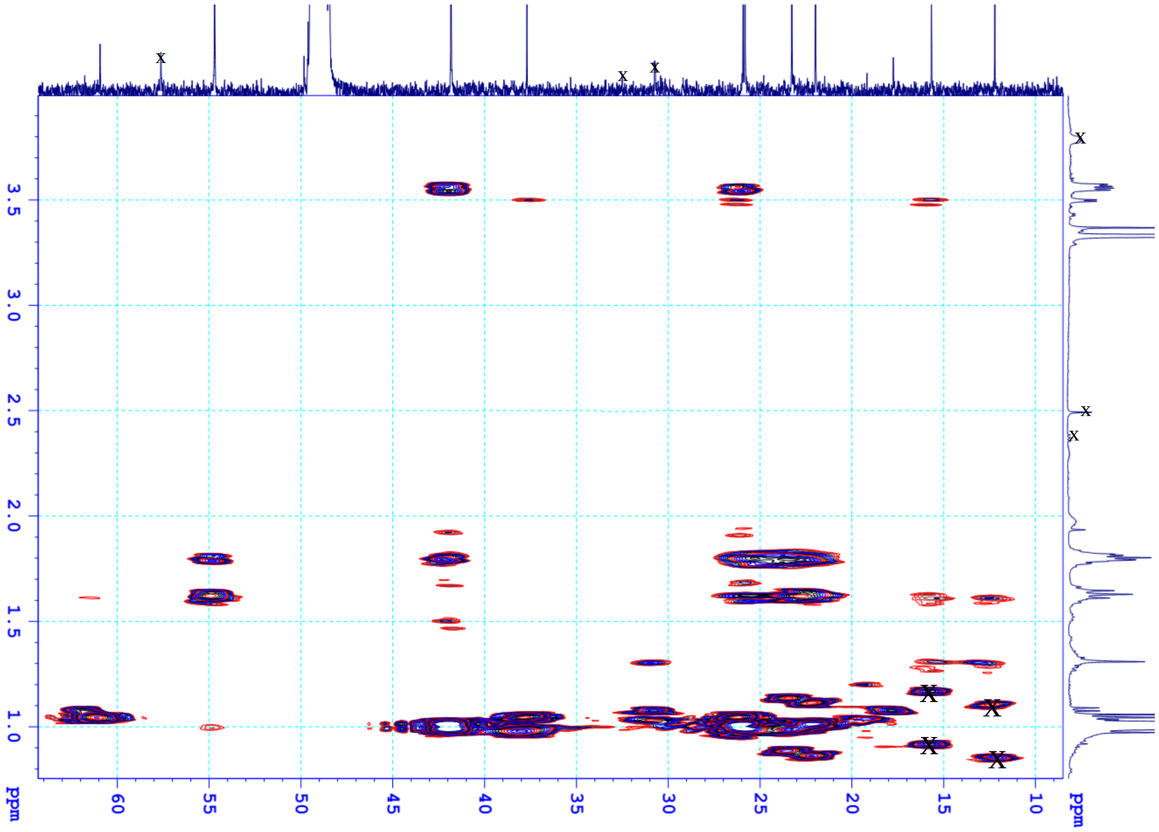

Figure S36. HMBC Spectrum of Compound **3** in CD_3OD (extension)

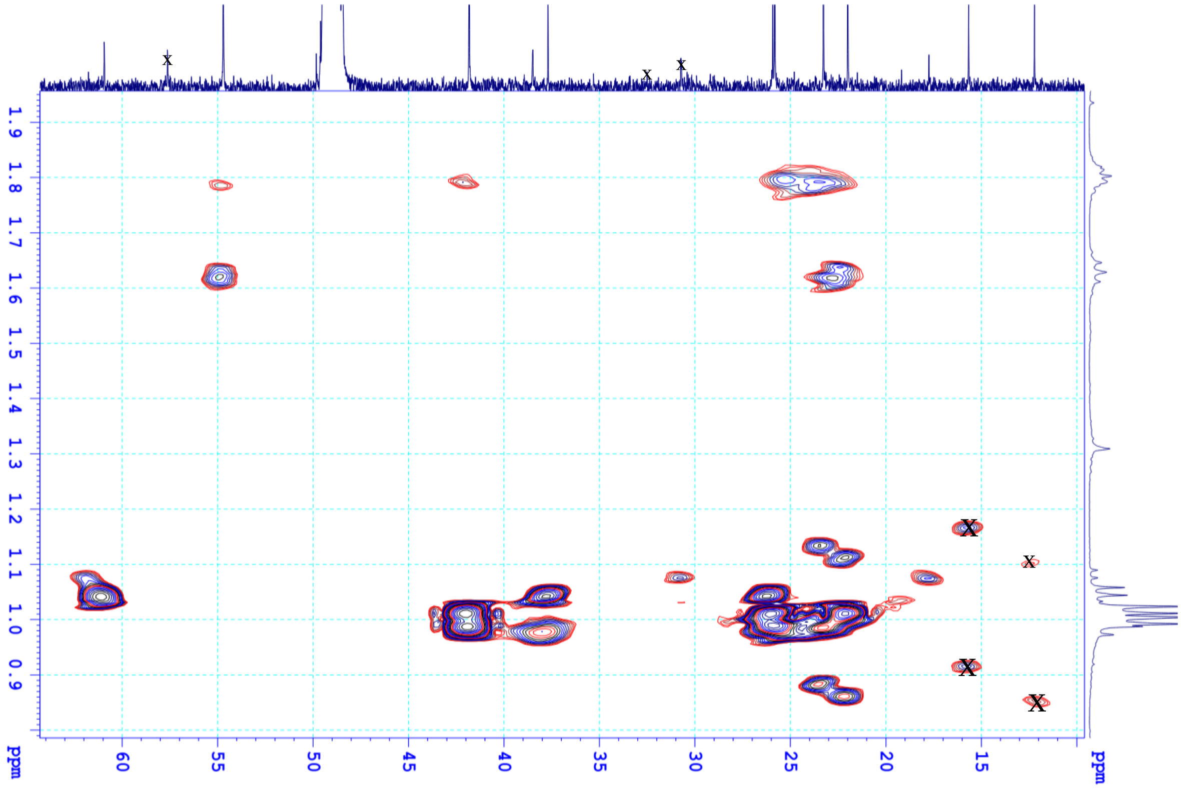

Figure S37. HMBC Spectrum of Compound **3** in CD_3OD (extension)
